# MIRA-EDCA: Multi-Indicator Responsive Adaptation of the Contention Window for Priority-Aware Safety Broadcasting in IEEE 802.11p Vehicular Networks

**DOI:** 10.3390/s26206476

**Published:** 2026-10-13

**Authors:** Amir Lazarev, Muhammad Sadiq, Hui Tang, Alisher Shakhobiddinov, Aihua Zhang, Kuanishbay Sadatdiynov, Ernazar Reypnazarov, Nurshod Akhmedov

**Affiliations:** 1 Department of Infocommunication Engineering, Tashkent University of Information Technologies Named After Muhammad al-Khwarizmi, Tashkent 100200, Uzbekistan; a.lazarev@tuit.uz (A.L.); n.axmedov@tuit.uz (N.A.); 2 Multimodal Intelligence and Distributed Trust Lab, Shenzhen University of Information Technology, Shenzhen 518172, China; sadiq@suit-sz.edu.cn; 3 School of Information and Communication, Shenzhen University of Information Technology, Shenzhen 518172, China; tangh@suit-sz.edu.cn; 4 Department of Television and Radio Broadcasting Systems, Tashkent University of Information Technologies Named After Muhammad al-Khwarizmi, Tashkent 100200, Uzbekistan; a.shakhobiddinov@tuit.uz; 5 Center for International Exchange and Cooperation, School of International Exchange, Shijiazhuang University, Shijiazhuang 050035, China; 6 Department of Data Transmission Networks and Systems, Nukus State Technical University, Nukus 230101, Uzbekistan; k.sadatdiynov@nukusstu.uz; 7 Department of Data Transmission Networks and Systems, Tashkent University of Information Technologies Named After Muhammad al-Khwarizmi, Tashkent 100200, Uzbekistan; e.reypnazarov@tuit.uz

**Keywords:** IEEE 802.11p, vehicular networks, V2V communication, EDCA, contention window adaptation, safety message broadcasting, channel busy ratio, backoff freeze ratio, priority-aware channel access

## Abstract

Reliable vehicular safety broadcasting requires channel access that limits collisions without imposing excessive delay on urgent messages. However, fixed contention windows and single-indicator adaptation do not fully represent the combined effects of contender population, channel occupancy, and repeated backoff interruption. This study proposes Multi-Indicator Responsive Adaptation integrated with Enhanced Distributed Channel Access (MIRA-EDCA), a deterministic contention-control mechanism that separates potential contention through a normalized contender indicator, realized channel occupancy through the Channel Busy Ratio (CBR), and node-level backoff interruption through the Backoff Freeze Ratio (BFR), and combines them through a bounded contention-pressure function. The measure drives class-specific contention-window updates through discrete levels and a one-level-per-epoch transition rule while preserving priority differentiation between event-driven and periodic safety traffic. MIRA-EDCA was evaluated in a controlled slot-level IEEE 802.11p simulation over 30 density–traffic operating points formed by ten vehicle-population levels and three event-load levels, with 30 Monte Carlo realizations per operating point. Relative to Standard EDCA, MIRA-EDCA reduced collision probability by 31.92% while increasing conditional mean event-message MAC access delay by 0.66%; for vehicle populations of 100 or more, collision reduction reached 33.40% with a delay increase below 1%. Under the implemented common-environment proxy comparisons, MIRA-EDCA exhibited an 88.6% lower conditional mean event-message MAC access delay than the Dynamic Contention Window common-environment proxy (DYCW-2023-CE) and a 31.7% lower conditional mean MAC access delay than MA-DRL-2026-CE-PS. The corresponding transmitter-side SBR difference relative to DYCW-2023-CE was +0.899 percentage points. These comparisons refer only to the proxy controllers implemented under the matched assumptions of this study and not to the complete source protocols. These results support the proposed MAC-layer contention-control logic within the evaluated IEEE 802.11p common-contention-domain abstraction; they characterize collision–access-delay behavior at the modeled MAC layer and do not constitute receiver-side or end-to-end reliability validation.

## 1. Introduction

Vehicular communication has become an important technological component of intelligent transportation systems (ITS), enabling vehicles to exchange information about their motion states, surrounding traffic conditions, and safety-related events. In vehicle-to-vehicle (V2V) communication, safety information is predominantly disseminated through broadcast transmission because the same message may be relevant to several neighboring vehicles simultaneously. The effectiveness of such communication depends not only on successful channel access but also on the time required to obtain that access. Consequently, reliability and latency are closely coupled requirements in vehicular safety broadcasting.

IEEE 802.11p was introduced to extend IEEE 802.11 medium access control (MAC) and physical-layer functionality to vehicular environments [1]. The IEEE 802.11 Working Group subsequently incorporated the amendment into later revisions of the IEEE 802.11 standard; nevertheless, the IEEE 802.11p channel-access model remains a widely used reference for the analysis of contention-based vehicular communication. In its contention-based operation, a vehicle senses the channel, observes the required arbitration interval, randomly selects a backoff counter from a contention window (CW), and decrements that counter while the medium remains idle. If the medium becomes busy, the countdown is suspended until contention can resume. This distributed procedure avoids centralized scheduling, but its performance becomes increasingly sensitive to the number of simultaneously contending vehicles.

The IEEE 802.11-based vehicular access technology has subsequently evolved toward IEEE 802.11bd, which extends the earlier IEEE 802.11p/Dedicated Short-Range Communications (DSRC) framework to support more demanding vehicle-to-everything (V2X) communication requirements while maintaining backward-oriented evolution of the technology [2,3,4,5]. These developments reinforce the continuing relevance of contention-based vehicular channel access and its reliability–latency behavior, even as the physical and MAC capabilities of vehicular radio systems evolve.

Safety broadcasting is particularly affected by this behavior. When the contention population is small, a relatively narrow CW provides rapid access because the expected backoff duration remains short. As the number of contenders increases, however, the probability that several vehicles select the same or closely aligned backoff states also increases. Concurrent transmission attempts then produce collisions and reduce successful message dissemination. Increasing the CW distributes transmission attempts over a larger backoff space and can reduce collisions, but it simultaneously increases channel-access delay. Therefore, CW control is not simply a collision-minimization problem; it involves a fundamental reliability–latency trade-off.

The problem is intensified by the characteristics of safety-message traffic. Periodic messages are generated continuously to provide updated vehicle-state information, whereas event-triggered messages may correspond to less frequent but more urgent situations. Adaptive beaconing research has further shown that the transmission behavior of periodic vehicular safety messages can be adjusted according to network density, channel conditions, and application requirements in order to mitigate channel congestion [6]. Previous work on Basic Safety Message dissemination has shown that collisions and dissemination delay can both become critical as vehicular contention grows [7]. Zhang et al. [7], for example, proposed a hybrid MAC protocol for safety-message broadcasting and demonstrated that contention and transmission coordination strongly affect delivery reliability and delay. The coexistence of periodic and event-driven information further implies that adaptive channel access should avoid protecting reliability at the cost of excessive latency for high-priority traffic.

Vehicular channel congestion has also been addressed through decentralized congestion-control mechanisms that adapt transmission behavior according to locally observed channel conditions [8,9,10,11,12]. Standardized and research-oriented decentralized congestion control (DCC) approaches use channel-load information to regulate parameters such as message generation rate, transmit power, or data rate, while algorithms such as LIMERIC demonstrate the effectiveness of distributed channel-load feedback for stabilizing vehicular broadcast traffic [8,9,10,11,12]. Previous studies have further shown that the control timescale and response dynamics are important because overly reactive adaptation may introduce oscillatory behavior, whereas slower adaptation can reduce responsiveness to changing traffic conditions [10,11].

One direction for addressing the limitations of fixed CW operation has been to derive the backoff configuration from network density or channel-load information. Yu et al. [13] developed an adaptive backoff scheme in which different vehicle densities are associated with an appropriate initial contention window, while a network busy factor is subsequently used to adjust the CW according to channel conditions. Their study demonstrates that both vehicle density and channel activity provide useful information for broadcast-oriented CW control. However, the adaptation remains closely coupled to the selected initial CW and busy-factor formulation.

Other approaches use locally observed communication outcomes as feedback. Chien and Giang [14] proposed an adaptive CW mechanism based on recently transmitted and received frames. Each vehicle estimates local congestion from the successful-frame reception rate and changes its CW while retaining EDCA-based priority differentiation. In a subsequent study, Chien and Giang [15] introduced an adaptive sliding contention-window mechanism for safety messages with different priority levels. Their mechanism modifies the CW using the percentage of successfully received frames and a dynamic persistence factor, and the reported simulations show reduced collision rates under dense traffic conditions.

Priority-aware contention adaptation has also been investigated using contextual information. Tripti and Jibukumar [16] considered beacon and event safety messages separately and proposed a context-aware MAC mechanism in which the CW of emergency traffic is adjusted according to a priority value derived from vehicular information. The study illustrates the importance of preserving service differentiation between routine and urgent safety traffic rather than treating all safety broadcasts identically. Such mechanisms improve traffic differentiation, but their control variables are primarily associated with packet context and application priority rather than with a direct decomposition of local MAC contention.

Density-driven CW selection provides another deterministic solution. Septa [17] proposed a dynamic contention-window mechanism in which vehicle density is mapped to different CW ranges. The method is combined with cooperative relay communication for safety-message dissemination, and the reported analytical and simulation results indicate that density-dependent CW adaptation can improve throughput and reduce delay and packet-drop behavior compared with a fixed configuration. The density-to-CW concept is attractive because of its simplicity and direct interpretation. However, density represents the number of potential contenders and does not necessarily indicate their instantaneous channel activity. Two contention domains containing the same number of vehicles may generate substantially different offered loads.

Recent analytical research has continued to investigate this relationship. Hota et al. [18] proposed an adaptive contention-window approach in which collision probability is related to the number of contending vehicles and compared with a threshold to determine an appropriate CW. Their numerical analysis evaluates collision probability, delay, throughput, and channel-busy behavior and reports substantial gains over conventional IEEE 802.11p operation. This work reinforces the value of density- and collision-aware contention control but also highlights that the selection of the observation variables remains central to the design of a deterministic adaptive CW mechanism.

Learning-based approaches provide an alternative mapping from contention observations to MAC decisions. Pressas et al. [19] applied Q-learning with collective contention estimation, Pei et al. [20] combined Q-learning-based CW adaptation with two-hop-neighbor information, and Kumar et al. [21] used Actor–Critic-based CW selection. More recent approaches include reinforcement-learning-based resource allocation under varying density and channel demand [22] and multi-agent deep-reinforcement-learning CW adaptation using locally observable MAC information [23]. These methods can represent nonlinear state–action relationships and combine multiple observations, but they require specification of the observation/action spaces, reward structure, and learning procedure.

Collision mitigation in vehicular MAC protocols is not limited to contention-window adaptation. Linn et al. [24] proposed a contention-free cooperative MAC protocol that uses vehicle and neighboring-node information, trajectory estimates, occupied and available transmission slots, and collision-resolution procedures to reduce heterogeneous collisions and access delay. This represents a different access-control philosophy in which collision suppression is achieved mainly through coordinated transmission opportunities rather than adaptive contention-window selection. Although such contention-free mechanisms are not directly equivalent to EDCA-based CW adaptation, they further demonstrate the close relationship between medium-access organization, collision probability, and channel-access latency in dynamic vehicular networks.

The representative approaches discussed above are summarized in Table 1. The purpose of the comparison is not to rank heterogeneous protocols using results reported under different experimental assumptions, but to identify the type of contention information used by each method and the corresponding channel-access adaptation philosophy.

Table 1 shows that the distinction of MIRA-EDCA does not arise from the isolated use of contender information, channel-load measurements, priority differentiation, or adaptive CW control. Density- and contender-based approaches [13,17,18,20] characterize potential competition for the medium, whereas feedback- and channel-state-based approaches [13,14,15,22] capture aspects of realized communication activity. Learning-based methods [19,20,21,22,23] can combine multiple observations through a learned policy. However, the representative CW-adaptation approaches do not explicitly organize potential contender population, aggregate channel occupancy, and node-level backoff interruption as three complementary inputs to a bounded deterministic contention-pressure measure.

These three observations represent related but non-equivalent aspects of contention. Vehicle or neighbor population indicates how many nodes may compete for access but does not directly describe their instantaneous activity. CBR measures aggregate medium occupancy but does not indicate whether that activity actually interrupts the backoff process of a particular contending node. BFR provides this node-level backoff-interruption information. The remaining control problem is therefore to combine these complementary observations while preserving traffic priority and preventing excessive contention-window variation.

### Novelty and Positioning of MIRA-EDCA

To address this gap, this paper proposes Multi-Indicator Responsive Adaptation (MIRA) for priority-aware vehicular safety broadcasting. Novelty is not claimed for the individual use of vehicle-population information, CBR, EDCA priority differentiation, or backoff freezing. The control-level contribution is their integration into a bounded deterministic contention-pressure representation in which potential contention, realized channel occupancy, and node-level backoff interruption are treated as complementary observations.

MIRA-EDCA translates this contention state into class-specific CW adaptation through predefined admissible levels and restricts the active CW to a one-level transition per adaptation epoch. Event-driven and periodic traffic retain their EDCA-based priority differentiation. The proposed architecture therefore combines multi-indicator contention estimation, bounded fusion, class-specific quantization, and constrained state transition without replacing the underlying priority-access mechanism.

At the control level, this architecture differs from representative deterministic CW-adaptation approaches [13,17,18] in the way the observed contention state is constructed and translated into an actuation decision. The method in [13] combines a density-derived initial CW with subsequent busy-factor adjustment, whereas [17] maps vehicle density to predefined CW ranges and [18] determines CW values from contender-dependent collision behavior and threshold comparison. In contrast, MIRA-EDCA explicitly separates potential contention, realized aggregate occupancy, and node-level backoff interruption before constructing a bounded contention-pressure state. This state is then passed through class-specific quantization and a one-level-per-epoch transition constraint. The resulting control increment is therefore not the introduction of a new individual indicator, but the separation of three contention phenomena, their bounded monotone fusion, and the rate-limited translation of the fused state into priority-preserving CW actuation.

This control architecture is defined through explicit verifiable design criteria rather than only through post hoc performance observations. The contention-pressure mapping is required to remain bounded, to be monotone with respect to increasing CBR or BFR, to retain the contribution of either realized-load indicator when the other is small, to reduce additive double counting as both indicators approach saturation, and to remain compatible with the normalized contender-dependent control input. The subsequent CW actuation is additionally constrained by predefined class-specific levels and by a maximum one-level transition per adaptation epoch. These properties are established analytically in Section 2.1.4 and are evaluated empirically through the ablation, correlation, and sensitivity analyses.

The proposed method is evaluated under a common slot-level simulation environment together with Standard EDCA, a density-driven reference derived from the published DYCW rule [17], and a learning-based reference derived from the recent MA-DRL direction [23]. The same exogenous traffic realizations and channel-access assumptions are used for the direct comparisons so that the observed differences can be associated with the corresponding contention-control mechanisms. Separate ablation and sensitivity experiments are used to distinguish the contribution of the individual indicators and to examine the influence of the main adaptation parameters.

The major contributions of this study are as follows:

1. A bounded multi-indicator contention-pressure formulation is developed for priority-aware vehicular safety broadcasting. The formulation explicitly separates potential contention through the normalized local contender indicator, realized aggregate channel occupancy through CBR, and node-level backoff interruption through BFR. Its control-level contribution is the bounded monotone fusion of these non-equivalent observations according to explicit design criteria, rather than the introduction of the individual indicators themselves.
2. A constrained class-specific contention-window adaptation procedure is proposed in which the raw contention response is mapped to predefined admissible contention-window levels for event-driven and periodic safety traffic. The active contention window is restricted to a one-level transition per adaptation epoch, while the existing priority differentiation between the two traffic classes is preserved. This design limits abrupt contention-window changes without replacing the underlying Enhanced Distributed Channel Access priority structure. Together with the bounded contention-pressure mapping, this quantization and transition constraint forms the actuation stage of the proposed control architecture.
3. The roles of the normalized local contender indicator, CBR, and BFR are evaluated through ablation. The Density-only, Joint-Load, and complete MIRA variants use the same quantization and CW-update rules, allowing the effect of the information supplied by each contention indicator to be isolated.
4. The proposed scheme is compared with fixed, density-driven, and learning-based contention control under a common evaluation protocol. Standard EDCA, DYCW-2023-CE, MIRA-EDCA, and the frozen MA-DRL-2026-CE-PS comparator are evaluated using paired Monte Carlo realizations and common random traffic inputs.
5. The sensitivity of the frozen proposed adaptation is examined independently of the primary evaluation. The analysis considers variation in the reference contender-count parameter, ±20% static bias in the local contender-count input, adaptation-epoch duration, and measurement horizon. These experiments characterize parameter dependence under the tested controlled perturbations; they are not used to claim robustness to delayed, temporally correlated, or burst-dependent measurement errors, nor to establish practical deployability.

## 2. Materials and Methods

### 2.1. MIRA-EDCA System Model and Adaptive Contention Mechanism

We consider distributed safety-message broadcasting among N vehicles sharing a common IEEE 802.11p-based wireless channel. The system is intended to represent a local V2V contention domain in which multiple vehicles independently attempt to access the medium using contention and backoff procedures. Two safety-message categories are considered: high-priority event-driven traffic and periodic vehicle-status traffic.

The modeled road segment has length L, and each vehicle has a nominal communication range R. In the present common-contention-domain abstraction, explicit vehicle coordinates and time-varying neighbor sets are not generated. Instead, the effective local population is represented by a deterministic geometric mean-contention proxy derived from the global population, road length, and nominal communication range. The effective number of vehicles within the contention domain is therefore approximated as

(1) $$N_{\mathrm{eff}}=\mathrm{round}\left[1+\left(N-1\right)\frac{2R}{L}\right]$$

The tagged vehicle is included in *N*_eff_. Therefore, the estimated number of neighboring contenders used to construct the first MIRA indicator is

(2) $$\hat{N}=N_{\mathrm{eff}}-1$$

For the baseline configuration,

*L* = 2000 m, *R* = 300 m (3)

and consequently $\frac{2R}{L}=0.3$. For example, global populations *N* = 20, 60, 100, and 200 correspond to approximately 7, 19, 31, and 61 vehicles in the effective local contention domain, respectively.

Accordingly, *N*_eff_ should not be interpreted as an instantaneous physical neighbor count or as the result of explicit vehicle placement. It represents the nominal local contention population used to maintain a controlled relationship between global vehicle population and the modeled common contention domain. Likewise, the resulting contender estimate used by MIRA is a deterministic nominal input for the primary evaluation rather than a time-varying neighbor-table measurement. Consequently, spatial clustering, vehicle motion, and temporal changes in neighborhood membership are not represented by Equations (1)–(3).

Channel access follows the contention and backoff principles associated with IEEE 802.11p/EDCA [1]. The channel is represented using a slot duration

*σ* = 13 μs (4)

Event-driven safety traffic is assigned the higher MAC priority and uses

*AIFSN_E_* = 2 (5)

whereas periodic traffic uses

*AIFSN_P_* = 3 (6)

With

*T_SIFS_* = 32 μs (7)

the corresponding arbitration intervals are

*AIFS_q_* = *T_SIFS_* + *AIFSN_q_σ* (8)

where *q* ∈ {*E*,*P*} identifies event-driven or periodic traffic.

The baseline contention-window state counts are

*W_E_*_,*base*_ = 4 (9)

and

*W_P_*_,*base*_ = 8 (10)

Throughout this study, W denotes the contention-window state count, i.e., the number of discrete backoff values available for random selection. This definition is distinguished from the conventional IEEE 802.11 contention-window parameter, which specifies the maximum backoff-counter index. The two representations are related by *W_conv_* = *W* − 1.

Thus, a state count *W* corresponds to the backoff set {0,1,…,*W* − 1}. For example, *W* = 4 represents the four backoff states {0,1,2,3} and corresponds to a conventional maximum CW index of 3, whereas *W* = 8 represents {0,1,…,7} and corresponds to a conventional maximum CW index of 7. Unless explicitly stated otherwise, all numerical window values reported for the proposed method and the benchmark methods in this study are CW state counts W, not conventional maximum-index CW values.

Accordingly, for a contention-window state count W, the initial backoff counter is selected according to

*B_q_*∼*U*{0,1,…,*W_q_* − 1} (11)

Accordingly, *W_E_* = 4 corresponds to backoff values {0,1,2,3}, whereas *W_P_* = 8 corresponds to {0,…,7}.

When the channel is idle and the corresponding AIFS requirement has been satisfied, the backoff counter is decremented. If the medium becomes busy, countdown progression is suspended. For vehicle *i*, the backoff process can be represented as

(12) $$B_{i}\left(k+1\right)=\{\begin{array}{c} B_{i}\left(k\right)-1,\;\mathrm{idle}\;\mathrm{channel}\;\mathrm{and}\;B_{i}\left(k\right)>0\\ B_{i}\left(k\right)\;\mathrm{busy}\;\mathrm{channel}\; \end{array}$$

A node becomes eligible for transmission when its residual backoff counter reaches zero after satisfying the corresponding AIFS requirement. All nodes that become eligible at the same modeled access instant are treated as initiating transmission simultaneously. If exactly one node transmits at that instant, the attempt is classified as successful. If two or more nodes transmit simultaneously, all participating attempts are classified as collided transmissions. The model does not apply receiver capture or partial decoding among simultaneous transmissions; therefore, no transmitter involved in such a collision is counted as successful.

The transmission duration used in the common simulation environment is

*T_tx_* = 254 μs (13)

The medium is treated as busy for the transmission interval together with the following SIFS period,

*T_busy_*_,*tx*_ = *T_tx_* + *T_SIFS_* = 286 μs (14)

The same modeled busy duration is applied after both a successful transmission and a collision. Thus, a collision involving multiple simultaneous transmitters occupies one common busy interval rather than a busy interval multiplied by the number of colliding nodes. With *T_Tx_* = 254 μs, SIFS = 32 μs and a 13 μs slot duration, the modeled busy interval is *T_busy_* = 286 μs, corresponding to 22 slots. During this interval, the backoff countdown of other pending contenders is frozen, and the busy interval contributes once to the channel-busy-time accumulation used for CBR calculation. After the modeled busy interval, which already includes the SIFS component, the affected pending contenders wait the remaining class-specific arbitration interval AIFSN_q_σ before backoff countdown resumes. For epoch-based CBR and BFR accounting, a busy interval that reaches the end of the current observation epoch is accumulated only up to that epoch boundary, after which the next observation interval starts with reset measurement counters. This is the fixed accounting convention used consistently for all compared methods.

After either a successful attempt or a collision, the corresponding transmitting packet leaves the modeled contention process. Collided broadcast packets are not retransmitted by an acknowledgement-triggered MAC mechanism. Safety messages are modeled as broadcasts, and no acknowledgement-triggered retransmission is applied. This allows the effect of contention-window adaptation on the initial broadcast-access process to be examined directly.

#### 2.1.1. Normalized Local Contender Indicator

The first MIRA indicator represents the potential size of the local contention population. Let $\hat{N}$ denote the local contender-count input. In the primary evaluation, $\hat{N}$ is obtained from the deterministic nominal population proxy defined by Equations (1)–(3), rather than from explicit vehicle positions or a time-varying neighbor table. MIRA itself only requires a local contender-count input and subsequently maps this quantity through the bounded normalized contender indicator

(15) $$\rho_{N}=\frac{\hat{N}}{\hat{N}+W_{\mathrm{ref}}}$$

where *W_ref_* is the reference contender-count parameter. The baseline configuration uses

*W_ref_* = 8 (16)

Equation (15) maps the local contender-count estimate to the interval 0 ≤ *ρ_N_* < 1, thereby preventing the contender-population contribution from increasing without bound as the number of neighboring contenders grows. Accordingly, $\hat{N}$ is treated as an input estimate, whereas *ρ_N_* is the first contention indicator used by MIRA. The deterministic construction of this input is specific to the present evaluation model and is not an intrinsic requirement of the MIRA formulation; a practical implementation could obtain the contender-count input from periodically received neighbor information or another local estimation procedure. Such a dynamic estimation process is not modeled in the present study.

#### 2.1.2. Channel Busy Ratio

The second indicator describes modeled medium occupancy within the adopted contention abstraction. For an observation interval of duration *T_obs_*, the Channel Busy Ratio is defined as

(17) $$C=\frac{T_{\mathrm{busy}}}{T_{\mathrm{obs}}}$$

where *T_busy_* is the cumulative modeled busy duration used for channel-access and backoff-freeze accounting within the adopted slot-level abstraction.

Therefore,

0 ≤ *C* ≤ 1 (18)

In the present model, accumulated busy time is based on the modeled busy intervals defined by the transmission/busy-period procedure described above. Each modeled busy interval comprises the transmission interval and the following SIFS period. A successful transmission and a collision each contribute one busy interval of the same modeled duration, irrespective of the number of simultaneously transmitting nodes involved in the collision; overlapping simultaneous transmissions are therefore not counted multiple times in the CBR calculation.

The baseline MIRA-EDCA configuration uses a 100 ms observation and adaptation epoch. This interval is treated as a pre-specified control timescale rather than an optimized parameter. Its purpose is to provide a compromise between measurement stability and adaptation responsiveness: the observation window should be sufficiently long to accumulate representative busy-channel and backoff-freeze observations, while remaining short enough for the contention window to respond to changes in the local traffic and contention state. In the adopted traffic model, 100 ms also corresponds to the nominal interval between periodic 10 Hz safety-message generations, providing a consistent timescale for aggregating contention observations and updating the controller. The value is therefore used as a balanced baseline operating point, not as a theoretically or empirically proven optimum.

Unlike the normalized local contender indicator *ρ_N_*, which represents the potential contention population, CBR reflects the modeled channel activity accumulated during the current measurement interval.

#### 2.1.3. Backoff Freeze Ratio

Aggregate channel occupancy does not explicitly describe how strongly busy-medium periods affect the backoff process of an individual node. For this reason, MIRA introduces the BFR as its third contention indicator.

Let *N_freeze_*_,*i*_ denote the number of backoff opportunities during which vehicle *i* is prevented from decrementing its counter because the channel is busy, and let *N_count_*_,*i*_ denote the number of opportunities during which the countdown successfully progresses. The BFR is defined as

(19) $$F_{i}=\frac{N_{\mathrm{freeze},i}}{N_{\mathrm{freeze},i}+N_{\mathrm{count},i}+\varepsilon }$$

where *ε* is a small positive constant used to avoid division by zero.

Thus,

0 ≤ *F_i_* ≤ 1 (20)

Accordingly, *F_i_* constitutes the third MIRA indicator: a larger value indicates persistent interruption of the local backoff process, whereas a smaller value indicates that the node can generally progress toward transmission.

#### 2.1.4. Multi-Indicator Contention Pressure

The three MIRA indicators provide complementary information about the local contention state. The normalized local contender indicator *ρ_N_* represents the potential contention population, the Channel Busy Ratio *C* represents realized aggregate medium occupancy, and the Backoff Freeze Ratio *F_i_* represents the extent to which medium activity interrupts the local backoff process of vehicle *i*. MIRA combines these three indicators through the contention-pressure function

*Ψ_i_* = *ρ_N_*(*C* + *F_i_* − *CF_i_*) (21)

Thus, *ρ_N_*, *C*, and *F_i_* are the three input indicators of MIRA, whereas *Ψ_i_* is their combined contention-pressure output used for CW adaptation.

The channel-load component

*G*(*C*,*F_i_*) = *C* + *F_i_* − *CF_i_* (22)

is bounded within

0 ≤ *G*(*C*,*F_i_*) ≤ 1 (23)

It increases when either CBR or BFR increases, while the interaction term −*CF_i_* prevents simple additive accumulation from exceeding unity.

Consequently,

0 ≤ *Ψ_i_* < 1 (24)

The fusion in (21) is designed to satisfy several control-oriented properties rather than to approximate a statistically fitted predictor. Let the channel-load component be written as *L_i_* = *C* + *F_i_* − *CF_i_* = 1 − (1 − *C*)(1 − *F_i_*). Because C,F*_i_* ∈ [0, 1], it follows directly that *L_i_* ∈ [0,1]. The function is monotone in both arguments, since $\frac{\partial L_{i}}{\partial C}=1-F_{i}\geq 0$, $\frac{\partial L_{i}}{\partial F_{i}}=1-C\geq 0$. Thus, an increase in either aggregate channel occupancy or local backoff interruption cannot decrease the estimated load component. The boundary conditions are also consistent with the intended interpretation: *L_i_*(*C*,0) = *C*, *L_i_*(0,*F_i_*) = *F_i_* and *L_i_*(*C*,1) = *L_i_*(1,*F_i_*) = 1. Therefore, either indicator can represent contention when the other is small, while saturation of either indicator drives the load component toward its upper bound.

The interaction term −*CF_i_* has an important control role. Since *L_i_* − *C* = *F_i_*(1 − *C*) ≥ 0, *L_i_* − *F_i_* = *C*(1 − *F_i_*) ≥ 0, the fusion retains the contribution of each indicator while adding only the unsaturated contribution of the other. At the same time, *L_i_* ≤ *C* + *F_i_*, so the two indicators are not simply accumulated additively. The mixed derivative $\frac{\partial^{2}L_{i}}{\partial C\partial F_{i}}=-1$ shows that the marginal contribution of one indicator decreases as the other approaches unity. This diminishing-interaction property limits double counting when both measurements respond to the same underlying contention increase.

The complete contention pressure is obtained by multiplying this bounded load component by the normalized local contender indicator, *Ψ_i_* = *ρ_N_L_i_*. Since 0 ≤ *ρ_N_* ≤ 1, the resulting contention pressure remains bounded, and 0 ≤ *Ψ_i_* ≤ *ρ_N_* ≤ 1.

The factor *ρ_N_* therefore acts as a population-dependent gate on the realized channel-load response: the same CBR/BFR observation produces a stronger contention-pressure response when the local contender population is larger, while isolated channel activity does not by itself produce an unbounded CW expansion. The partial derivatives $\frac{\partial \Psi_{i}}{\partial C}=\rho_{N}(1-F_{i})$, $\frac{\partial \Psi_{i}}{\partial F_{i}}=\rho_{N}(1-C)$ also show that the response remains monotone but naturally saturates as either realized-load indicator becomes large.

This formulation should not be interpreted as assuming statistical independence between CBR and BFR. Although 1 − (1 − *C*)(1 − *F_i_*) has the same algebraic form as a probabilistic union operator, it is used here only as a bounded deterministic aggregation rule. The correlation analysis in Section 3.8 shows that CBR and BFR are related but non-redundant observations: their pooled association is positive, whereas the within-operating-point residual association is weak after controlling for the common density–traffic condition.

Accordingly, the mathematical fusion was selected to satisfy five explicit design criteria: boundedness, monotonicity, retention of either indicator when the other is small, diminishing double counting when both indicators are high, and compatibility with the normalized contender-dependent CW control. No empirically fitted weighting coefficients are introduced, and the formulation is not claimed to be statistically optimal. Its purpose is to provide a transparent deterministic mapping from complementary contention observations to a bounded control variable.

#### 2.1.5. Contention-Window Adaptation

For traffic class *q*, the raw MIRA-EDCA contention-window response is calculated as

(25) $$W_{q,i}^{\mathrm{raw}}=\frac{W_{q,\mathrm{base}}}{1-\Psi_{i}+\varepsilon }$$

As *Ψ_i_* increases, the raw contention window becomes larger, reducing the probability that several nodes select closely aligned backoff states.

Direct use of (25), however, could produce frequent small changes in the active contention window. The raw value is therefore quantized to predefined admissible levels.

For event-driven safety traffic,

*W_E_* = {4, 8, 16, 32, 64, 128} (26)

whereas for periodic traffic,

*W_P_* = {8, 16, 32, 64, 128} (27)

Let $Q_{q}\left(W_{q,i}^{\mathrm{raw}}\right)$ denote mapping to the nearest admissible class-specific level. The requested contention-window state is

(28) $$W_{q,i}^{*}=Q_{q}\left(W_{q,i}^{\mathrm{raw}}\right)$$

To limit rapid oscillations, the active CW is allowed to change by at most one quantization level during a single adaptation epoch. If 𝓁*_q_*(*k*) denotes the index of the active CW level during epoch *k*, then

(29) $$l_{q}\left(k+1\right)=l_{q}\left(k\right)+\mathrm{clip}\left[l_{q}^{*}\left(k\right)-l_{q}\left(k\right),-1,1\right]$$

where $l_{q}^{*}\left(k\right)$ is the level requested by (28).

The updated contention window is subsequently

*W_q_*(*k* + 1) = *W_q_*[𝓁*_q_*(*k* + 1)] (30)

This one-level-per-epoch rule prevents a short-term fluctuation in the indicators from immediately moving the MAC configuration across several contention-window levels.

#### 2.1.6. Priority-Aware Safety Traffic

Two simultaneous traffic processes are considered. Periodic safety messages are generated at

*f_P_* = 10 Hz (31)

corresponding to a nominal generation interval of

*T_P_* = 100 ms (32)

To avoid artificial synchronization of periodic transmitters, the first generation time of vehicle *i* is determined from an independent random phase

*ϕ_i_*∼*U*(0,*T_P_*) (33)

Subsequent periodic messages are generated at

(34) $$t_{i,n}^{\left(P\right)}=\phi_{i}+nT_{P}$$

Event-driven messages are generated according to a two-component hyperexponential model. For each event inter-arrival time, one of two exponential components is selected using

*p*_1_ = 0.8, *p*_2_ = 0.2 (35)

with nominal rates

*λ*_1_ = 0.5 s^−1^ (36)

and

*λ*_2_ = 5 s^−1^ (37)

The event inter-arrival distribution is therefore

(38) $$f_{T_{E}}\left(t\right)=p_{1}\lambda_{1}e^{-\lambda_{1}t}+p_{2}\lambda_{2}e^{-\lambda_{2}t},\;t\geq 0$$

The nominal mean event inter-arrival time is

(39) $$E\left[T_{E}\right]=\frac{p_{1}}{\lambda_{1}}+\frac{p_{2}}{\lambda_{2}}=1.64\;s$$

corresponding to approximately

(40) $$\frac{0.610\mathrm{events}}{s}$$

per vehicle.

To evaluate increasingly demanding event traffic, both component rates are multiplied by the event scaling factor

*s_E_* ∈ {1, 3, 5} (41)

Hence,

(42) $$\lambda_{m}^{\left(s\right)}=s_{E}\lambda_{m}$$

These cases are subsequently denoted as *EventScale* = 1, 3, and 5.

Each vehicle maintains at most one pending safety message. Event-driven traffic has strict application-level priority over periodic traffic. If an event message arrives while a periodic packet is pending, the event message replaces the periodic packet. A periodic arrival cannot replace an already pending event packet.

For arrivals belonging to the same class, the latest message replaces the previous pending message. This replacement policy is used because a more recent vehicle-state or event update can supersede stale information that has not yet obtained channel access.

Both traffic classes use a validity limit of

*D_max_* = 50 ms (43)

A packet that exceeds its validity interval before successful transmission is removed from the pending state.

### 2.2. Proposed MIRA-EDCA Operational Procedure

The mathematical formulation introduced in Section 2.1 defines the individual indicators and the contention-window response of MIRA-EDCA. This subsection summarizes their interaction as a complete distributed channel-access procedure. The principal objective of the proposed mechanism is to adjust the contention window according to the current contention state while preserving the access differentiation between event-driven and periodic safety traffic.

At the beginning of operation, each vehicle uses the corresponding baseline contention-window state count, *W_E_* = *W_E_*_,*base*_ = 4, for event-driven traffic and *W_P_* = *W_P_*_,*base*_ = 8 for periodic traffic. During each adaptation epoch, a vehicle collects the quantities required to construct the three MIRA indicators. The local contender-count estimate $\hat{N}$ is first transformed into the normalized local contender indicator *ρ*_N_ according to Equation (15). In parallel, cumulative channel-busy time is converted into the Channel Busy Ratio *C* according to Equation (17), while frozen and successfully decremented backoff opportunities are used to obtain the Backoff Freeze Ratio *F_i_* according to Equation (19). At the end of the observation epoch, the three MIRA indicators *ρ*_N_, *C*, and *F_i_* are combined using the contention-pressure function in Equation (21). The resulting value represents the contention state experienced by the vehicle during the completed observation interval. A low value of Ψ*_i_* retains the contention window near its class-specific baseline, whereas increasing contention pressure causes the requested CW to move toward larger admissible values. For each traffic class *q* ∈ {*E*,*P*}, the corresponding raw contention-window request is obtained from Equation (25). The raw value is not used directly. Instead, it is mapped to the nearest admissible level of the corresponding class-specific set (26) and (27). This quantization limits the number of possible MAC configurations and makes the adaptation behavior directly interpretable.

A second control mechanism limits the rate at which the active contention window can change. Even if the raw response requests a CW several levels above or below the current value, MIRA-EDCA moves by at most one level during one adaptation epoch, as defined in Equation (29). Consequently, the controller does not immediately jump from a small contention window to the largest available value after a short-term change in channel conditions.

The adaptation process does not modify the priority relation between the two safety-message classes. Event-driven traffic continues to use (5), whereas periodic traffic uses (6).

Thus, MIRA-EDCA adapts the backoff space while preserving the shorter arbitration interval assigned to high-priority event messages.

After the active contention windows have been updated, the observation counters used to calculate CBR and BFR are reset, and a new observation epoch begins. Previously selected backoff counters are not retrospectively redrawn merely because the adaptation state changes; the updated contention-window configuration applies when subsequent packet admissions require a new backoff selection.

The complete procedure is summarized in Algorithm 1.
**Algorithm 1.** Proposed MIRA-EDCA Contention-Window Adaptation**Input:** local contender estimate $\;\hat{N}$; reference contender-count parameter *W_ref_*; observation interval *T_obs_*; small positive constant ε; class-specific base windows *W_E_*_,*base*_ and *W_P_*_,*base*_; admissible CW sets 𝒲*_E_* and 𝒲*_P_*. **Output:** updated class-specific contention windows *W_E_*_,*i*_ and *W_P_*_,*i*_. 1: Initialize $W_{E,i}^{\left(o\right)}\leftarrow \;W_{E,\mathrm{base}}$ and $W_{P,i}^{\left(o\right)}\leftarrow \;W_{P,\mathrm{base}}$
2: for each adaptation epoch *k* = 0, 1, 2,… do 3: Obtain the local contender-count estimate $\hat{N}$
4: Measure cumulative busy-channel time *T_busy_* 5: Accumulate *N*_freeze,I_ and *N*_count,I_ during backoff operation 6: Compute the normalized local contender indicator $\;\;\rho_{N}\;\leftarrow \frac{\hat{N}}{\hat{N}+W_{\mathrm{ref}}}$
7: Compute the Channel Busy Ratio *C* $\;\;C\;\leftarrow \frac{T_{\mathrm{busy}}}{T_{\mathrm{obs}}}$
8: Compute Backoff Freeze Ratio *F_i_* $\;\;F_{i}\leftarrow \frac{N_{\mathrm{freeze},i}}{N_{\mathrm{freeze},i}+N_{\mathrm{count},i}+\varepsilon }$
9: Compute the MIRA contention pressure Ψ*_i_*               Ψ*_i_* ← *ρ_N_*(*C* + *F_i_* − *CF_i_*) 10: for each traffic class *q* ∈ {*E*,*P*} do 11: Compute the raw contention-window request $\;\;W_{q,i}^{\mathrm{raw}}\leftarrow \frac{W_{q,\mathrm{base}}}{1-\Psi_{i}+\varepsilon }$
12: Quantize the raw request to the nearest admissible class-specific CW level $\;\;W_{q,i}^{\mathrm{target}}\leftarrow Q_{q}\left(W_{q,i}^{\mathrm{raw}}\right)$
13: Determine the current and target CW-level indices $\;\;l_{\mathrm{current}}\leftarrow {\mathrm{level}}_{q}\left(W_{q,i}^{\left(k\right)}\right)$ $\;\;l_{\mathrm{target}}\leftarrow {\mathrm{level}}_{q}\left(W_{q,i}^{\mathrm{target}}\right)$
14: Limit the transition to one admissible CW level           𝓁*_next_* ← 𝓁*_current_* + clip(𝓁*_target_* − 𝓁*_current_*,−1,1) 15: Update the active contention window $\;\;W_{q,i}^{\left(k+1\right)}\leftarrow W_{q}\left[l_{\mathrm{next}}\right]$
16: end for 17: Retain *AIFSN_E_* = 2 and *AIFSN_P_* = 3 18: Reset the observation variables             *T_busy_* ← 0, *N*_freeze,i_ ← 0, *N*_count,i_ ← 0 19: end for

Here, *level_q_*(*W*) denotes the index of W within the ordered admissible CW set 𝒲*_q_* and clip(*x*,−1,1) = min{1,max{−1,x}}.

**Figure 1:**
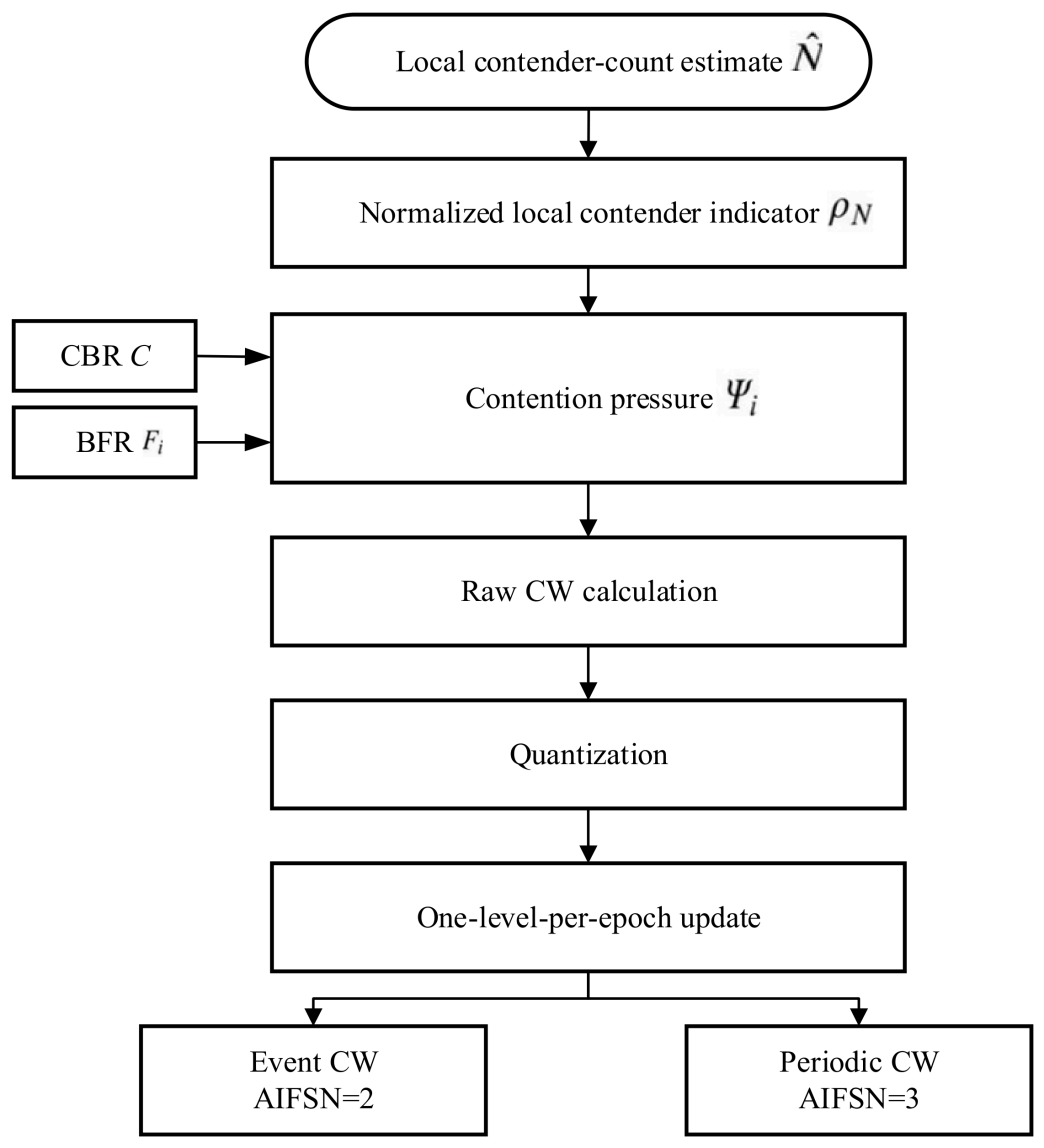
Operational flow of the proposed MIRA-EDCA contention-window adaptation mechanism.

The operational sequence of the proposed MIRA-EDCA mechanism is illustrated in Figure 1.

As shown in Figure 1, MIRA-EDCA separates contention estimation from the subsequent contention-window update. The local contender-count estimate $\hat{N}$ is first transformed into the normalized local contender indicator *ρ_N_*, which represents the potential contention population. In parallel, the Channel Busy Ratio *C* represents observed aggregate channel occupancy, while the Backoff Freeze Ratio *F_i_* reflects the extent to which medium activity interrupts the local backoff process of vehicle *i*. The three MIRA indicators *ρ_N_*, *C*, and *F_i_* are then combined into the bounded contention-pressure measure Ψ*_i_*, which controls the raw CW calculation.

The calculated CW is not applied directly. It is first mapped to the nearest admissible class-specific contention-window level, after which the active CW can move by no more than one level during a single adaptation epoch. This restriction prevents abrupt changes in the backoff space and limits excessive CW expansion in response to short-term fluctuations. The updated values are then applied separately to event-driven and periodic traffic, while the corresponding *AIFSN_E_* = 2 and *AIFSN_P_* = 3 settings are retained. Consequently, the adaptation modifies the contention intensity without removing the priority differentiation between the two safety-message classes.

#### Practical Measurement Feasibility of the MIRA Indicators

The three quantities required by MIRA can be obtained from locally available MAC/PHY observations and do not inherently require additional control-packet exchange. The practical implementation, however, requires appropriate measurement counters and exposes several estimation uncertainties that are abstracted in the primary simulation model.

The local contender-count estimate $\hat{N}$ can be approximated from an active-neighbor table constructed from successfully decoded periodic safety broadcasts. A node may count distinct recently observed transmitters within a sliding observation interval and remove entries that have not been observed for a predefined aging period. This quantity should be interpreted as a practical proxy for the active local contention population rather than an exact count of all physical neighbors. Packet losses, temporary channel blockage, or unequal transmission activity may cause underestimation, whereas stale neighbor entries may cause overestimation. The primary evaluation in this study uses the effective local contender population directly, while the separate estimation-error experiment examines the sensitivity of MIRA to moderate deviations from this nominal estimate.

CBR can be obtained directly from the physical carrier-sensing state already required by CSMA/CA operation. During each observation epoch, the node accumulates the duration for which the Clear Channel Assessment state indicates that the medium is busy and normalizes this duration by the observation interval. This measurement is passive and requires only a local busy-time accumulator; it does not introduce additional over-the-air signaling.

BFR can likewise be measured locally within the MAC backoff procedure. A backoff opportunity is counted as a freeze event when a pending transmission has an active backoff counter but the counter cannot decrement because the medium is sensed as busy. A countdown event is recorded when the counter is permitted to decrement. Therefore, implementation requires two local MAC counters for freeze and successful countdown opportunities during each adaptation epoch. These counters may be maintained in the MAC implementation or exposed through driver/firmware instrumentation, but their collection does not require additional transmitted packets.

Consequently, the communication overhead of MIRA is limited because CBR and BFR rely on passive channel-access observations, while contender estimation can reuse information already contained in received safety broadcasts. The additional processing state consists mainly of an active-neighbor table, a busy-time accumulator, two backoff-event counters, and the periodic contention-window update. The present study evaluates the control logic rather than a specific hardware or driver implementation; therefore, platform-dependent counter access and processing cost remain implementation considerations rather than experimentally measured quantities.

In a practical implementation, the locally observed indicators may also be delayed, biased, or noisy. The contender-count estimate may lag behind rapid topology changes because neighbor-table entries are accumulated and aged over a finite observation interval. Packet losses may produce temporary underestimation, whereas stale entries may cause positive bias. CBR may fluctuate because of short-term traffic bursts and the finite measurement interval, while BFR may exhibit similar short-term variability when only a small number of backoff opportunities is observed during an epoch. Such measurement imperfections can cause the contention state used by MIRA to differ from the instantaneous physical state of the channel. The bounded indicator formulation, discrete CW levels, and one-level-per-epoch transition rule limit the magnitude of a single CW response to these fluctuations, but they do not eliminate estimation error.

The sensitivity experiment reported in Section 3.7 addresses one limited aspect of this issue by perturbing the local contender-count estimate by ±20% relative to its nominal value. This test quantifies sensitivity to moderate systematic underestimation and overestimation of the contender population. It should not, however, be interpreted as a complete model of practical measurement uncertainty, because it does not reproduce time-correlated estimation errors, delayed neighbor-table updates, burst-dependent CBR noise, or uncertainty in the BFR counters. These effects require additional validation under time-varying measurement, mobility, and channel conditions, and are therefore treated as limitations and subjects for future work.

### 2.3. Simulation Parameters and Evaluation Settings

Based on the system model described above, the principal simulation parameters are summarized in Table 2. Unless otherwise stated, the same common configuration is used for Standard EDCA, MIRA-EDCA, and the common-environment benchmark methods.

No explicit vehicle mobility trajectory or speed process is generated in the present evaluation; vehicle motion and mobility-driven changes in neighborhood membership are outside the controlled common-contention-domain abstraction.

The Cartesian combination of ten vehicle-population levels and three EventScale values defines 10 × 3 = 30 density–traffic operating points in the primary two-factor evaluation grid. Road length, communication range, traffic-generation structure, PHY/MAC timing configuration, packet transmission duration, message-validity deadline, and periodic message rate are held fixed across this primary grid. Each operating point is evaluated using 30 Monte Carlo realizations; therefore, each method contributes 30 × 30 = 900 realizations to the primary evaluation.

The simulation model is intentionally scoped as a controlled slot-level MAC-layer contention environment designed to isolate the effect of contention-window adaptation under matched channel-access conditions. All vehicles within the effective contention domain are assumed to participate in a common observable channel-access process. Consequently, the model does not explicitly represent hidden-terminal relationships [25], mobility-dependent topology, distance-dependent propagation loss, time-varying fading, receiver capture, external interference, detailed physical-layer decoding, or receiver-specific packet reception. Within this abstraction, collision probability denotes simultaneous modeled MAC access attempts in the common contention domain, while the reported delay denotes MAC access delay. The Successful Broadcast Ratio is a transmitter-side metric indicating successful completion of the modeled contention and transmission process; it is not a receiver-side Packet Delivery Ratio. Accordingly, the reported metrics characterize MAC-layer contention-control behavior under the stated assumptions and should not be interpreted as end-to-end communication reliability or deployment-level vehicular-network performance.

The primary evaluation uses a 15 s measurement interval followed by a 50 ms drain period for packets generated near the end of the measurement horizon. With the baseline 100 ms adaptation epoch, each primary realization therefore contains 150 adaptation epochs. Performance statistics associated with generated safety traffic are retained according to the measurement and validity rules described above. Because a finite observation horizon may influence the estimated mean performance metrics, measurement-duration sensitivity is evaluated separately using longer realizations, as described in Section 2.6.

### 2.4. Implementation and Reproducibility Details

The simulation framework was implemented in MATLAB R2024a (The MathWorks, Inc., Natick, MA, USA). Channel access is simulated at slot-level resolution, including message generation, application-level priority replacement, class-specific AIFS behavior, random backoff selection, backoff countdown, backoff freezing, simultaneous-access collision detection, contention-window adaptation, packet expiration, and metric collection.

A common-random-number design is used for direct method comparison. For a given density, event-load condition, and Monte Carlo realization, all compared methods receive the same exogenous periodic and event traffic realization.

In addition, the same per-arrival uniform random variable is used to derive the initial backoff selection under the active contention window. If *U*∼*U*(0,1) denotes this common random variable, a backoff value for window W can be represented as

*B* = ⌊*UW*⌋ (44)

Thus, two compared schemes may use different CW values while remaining coupled to the same underlying random draw.

For the main evaluation, operating-point seeds are generated from the frozen base value

*S*_0_ = 710,000 (45)

The operating-point-specific seed is defined as

*S* = 710,000 + 10,000(*s* − 1) + 100*i_N_* + *m* (46)

where *s* identifies the EventScale condition, *i_N_* identifies the vehicle-population point, and m is the Monte Carlo realization index.

This deterministic construction allows every reported realization to be regenerated from its density, event-load condition, and Monte Carlo index.

The final primary evaluation was frozen before the additional ablation and sensitivity analyses were interpreted. No changes were subsequently made to the MIRA-EDCA contention-pressure formulation, *W_ref_*, admissible CW levels, or baseline 100 ms adaptation epoch on the basis of the final comparison results.

The MATLAB output is stored at two levels. Per-realization datasets retain operating-point identifier, method, seed, and metric information, whereas aggregated result files contain operating-point-level means and 95% confidence intervals. Paired-difference files are maintained separately for direct method comparisons.

### 2.5. Benchmark Methods and Statistical Evaluation Protocol

The primary comparison considers four channel-access configurations:

1. Standard EDCA;
2. DYCW-2023-CE;
3. MIRA-EDCA;
4. MA-DRL-2026-CE-PS.

Standard EDCA provides the fixed contention-window reference and uses

*W_E_* = 4,*W_P_* = 8 (47)

throughout the evaluation.

DYCW-2023-CE is based on the published dynamic contention-window rule in [17]. The source rule selects the CW according to the surrounding vehicle population as

(48) $$W_{\mathrm{DYCW}}=\{\begin{array}{c} 64,0<N_{\mathrm{DYCW}}\leq 15\\ 128,15<N_{\mathrm{DYCW}}\leq 30\\ 512,30<N_{\mathrm{DYCW}}\leq 100 \end{array}$$

Within the common evaluation environment,

*N_DYCW_* = *N_eff_* (49)

where *N_eff_* is obtained from Equation (1).

Only the published density-to-CW control rule is used in the common-environment comparator. The designation DYCW-2023-CE is therefore used to distinguish this benchmark from the complete cooperative communication architecture proposed in [17].

The learning-based comparator is denoted MA-DRL-2026-CE-PS, where CE indicates common-environment evaluation and PS denotes parameter sharing. Its design follows the recent learning-driven CW adaptation direction represented by [23]. A parameter-shared actor receives the observation vector

(50) $$o_{i}=\left[Q_{i},C_{i},r_{\mathrm{coll},i},{\bar{D}}_{i},\frac{W_{E,i}}{128}\right]$$

where *Q_i_* represents the pending-message state, *C_i_* is CBR, *r_coll_*_,*i*_ is the observed collision-related state, ${\bar{D}}_{i}$ is normalized access delay, and the final component represents the normalized event CW.

The actor contains three hidden fully connected layers with 128 units per layer and produces a continuous action, subsequently mapped to the admissible common-environment CW range.

Training uses an Actor–Critic formulation with learning rate 3 × 10^−4^, discount factor *γ* = 0.99, batch size 32, and replay capacity 100,000.

The final actor is frozen before the main held-out comparison. During evaluation, exploration is disabled and no online weight update is performed. The label MA-DRL-2026-CE-PS is used because several implementation details required for exact reproduction of the source method are not completely specified in the available description; therefore, the comparator should be interpreted as a common-environment learning reference rather than an exact reproduction of [23].

The benchmark implementations are used as common-environment comparison proxies rather than as complete reproductions of the corresponding source systems. For DYCW-2023-CE, only the published density-to-contention-window mapping is transferred to the common simulation environment; cooperative relay operation, acknowledgement-related procedures, and other protocol components of the original architecture are not reproduced. For MA-DRL-2026-CE-PS, the learning-based contention-window control principle is represented using a frozen parameter-shared Actor–Critic implementation under the same traffic and channel-access assumptions as the other methods. Because several implementation details required for exact source-level reproduction are not fully specified, this comparator is not presented as an exact replication of the complete method in [23]. Therefore, the numerical comparisons reported in this study should be interpreted as controlled comparisons of contention-window control strategies under matched simulation assumptions, rather than as evidence of general superiority over the complete DYCW or MA-DRL systems.

As summarized in Table 3, the benchmark implementations are intended to provide controlled references for fixed, density-driven, and learning-based contention-window control under matched simulation assumptions. The resulting comparisons therefore isolate differences in contention-control behavior within the common evaluation environment. They should not be interpreted as direct performance rankings of the complete source protocols or systems from which the comparator concepts were derived.

The omissions and common-environment adaptations listed in Table 3 may influence the numerical behavior of the proxy controllers relative to their complete source implementations. However, the present evaluation does not provide a basis for concluding that these differences systematically favor or disadvantage MIRA-EDCA. For DYCW-2023-CE, omitting cooperative communication and other source-specific protocol components may alter both contention and delay behavior in ways that cannot be inferred from the transferred density-to-CW rule alone. Likewise, the parameter-shared Actor–Critic implementation used for MA-DRL-2026-CE-PS may differ from the complete source method in observation structure, training dynamics, policy architecture, or other implementation details that are not sufficiently specified for exact reproduction. Therefore, all percentage differences involving these two benchmarks are interpreted only as differences between the implemented proxy controllers under the matched assumptions of the present study.

The primary performance metrics are collision probability, transmitter-side event-message successful broadcast ratio, event-message MAC access delay, CBR, and mean active event CW state count.

Collision probability is defined as

(51) $$P_{\mathrm{collision}}=\frac{N_{\mathrm{collided}\;\mathrm{attempts}}}{N_{\mathrm{total}\;\mathrm{transmission}\;\mathrm{attempts}}}$$

Here, collision probability is defined on a transmission-attempt basis rather than on a collision-event basis. If *m* ≥ 2 nodes transmit at the same modeled access instant, all *m* transmission attempts are counted as collided attempts. Thus, such an event contributes *m* attempts to the total-attempt count and m collided attempts to the collision numerator, while the channel occupancy generated by those simultaneous transmissions is represented by a single common busy interval.

Event-message Successful Broadcast Ratio is defined as

(52) $${\mathrm{SBR}}_{E}=\frac{N_{\mathrm{successful},\;E}}{N_{\mathrm{generated},\;E}}$$

The SBR used in this study is a transmitter-side successful-broadcast metric. It characterizes whether a generated message successfully passes the modeled contention and transmission process and should not be interpreted as receiver-specific Packet Delivery Ratio.

For a successfully transmitted event packet *j*, MAC access delay is

*D_E_*_,*j*_ = *t_j_*_,*success*_ − *t_j_*_,*generation*_ (53)

Event-message MAC access delay is evaluated conditionally on successful transmission. For each Monte Carlo realization, the successful event-message delay samples are summarized by their mean, median, and 95th percentile. The 95th percentile is obtained from the empirical delay samples using linear interpolation. Operating-point values reported in the Results are obtained by averaging the corresponding per-realization summaries over the 30 Monte Carlo realizations. Unsuccessful event messages are not assigned artificial or truncated delay values; their outcomes are quantified separately as described below.

To complement the conditional delay statistics, every generated event message is assigned to one terminal outcome: successful transmission, collision loss, expiration, replacement by a newer event message at the same node, or residual pending state at the end of the measurement-plus-drain interval. Accordingly, the event-message accounting satisfies *N_gen_*_,*E*_ = *N_succ_*_,*E*_ + *N_coll_*_,*E*_ + *N_exp_*_,*E*_ + *N_repl_*_,*E*_ + *N_res_*_,*E*_. Here, *N_coll_*_,*E*_ denotes event messages removed after a modeled simultaneous-access collision, *N_exp_*_,*E*_ denotes pending event messages whose age exceeds the 50 ms validity deadline, *N_repl_*_,*E*_ denotes pending event messages replaced by a newly generated event message at the same node, and *N_res_*_,*E*_ denotes event messages still pending after the measurement interval and the 50 ms drain period.

The event-message expiration, replacement, collision-loss, and incomplete-transmission rates are normalized by the number of generated event messages: *R_exp_* = *N_exp_*_,*E*_/*N_gen_*_,*E*_, *R_repl_* = *N_repl_*_,*E*_/*N_gen_*_,*E*_, *R_coll_* = *N_coll_*_,*E*_/*N_gen_*_,*E*_ and $R_{\mathrm{inc}}=\frac{\left(N_{\mathrm{gen},E}-N_{\mathrm{succ},E}\right)}{N_{\mathrm{gen},E}}=1-{\mathrm{SBR}}_{E}$. The residual rate is defined analogously as *R_res_* = *N_res_*_,*E*_/*N_gen_*_,*E*_. Hence, the incomplete-transmission rate captures all event messages that do not complete successfully, whereas the individual outcome rates identify the mechanism responsible for that incompletion.

For descriptive presentation, each method-specific operating-point mean is accompanied by a 95% confidence interval calculated across the 30 Monte Carlo realizations. These descriptive confidence intervals are not used as the criterion for declaring between-method statistical significance. Because all compared methods are evaluated using matched traffic realizations and matched random seeds, inferential comparisons are performed on paired Monte Carlo outcomes.

For methods *A* and *B*, let

*d_m_* = *x_A_*_,*m*_ − *x_B_*_,*m*_ (54)

where *m* = 1,…,30 denotes a matched Monte Carlo realization for the same operating point. The paired Student’s *t*-test statistic is:

(55) $$t=\frac{\bar{d}}{s_{d}/\sqrt{n}},\;n=30,\;\mathrm{df}=n-1=29$$

where $\bar{d}$—mean paired difference; *s_d_*—standard deviation of the paired differences.

For each metric and operating point, a two-sided paired Student’s *t*-test is applied to the 30 matched differences. The null hypothesis is *H*_0_:*E*[*d_m_*] = 0. Because the same method comparison is evaluated at 30 density–traffic operating points, the resulting 30 raw *p*-values are adjusted using the Holm–Bonferroni procedure. The correction is applied separately for each method-pair × metric family. An operating-point-level difference is classified as statistically significant only when the Holm-adjusted *p*-value is below 0.05.

The paired testing procedure is applied to collision probability, transmitter-side event-message SBR, incomplete-transmission rate, expiration rate, replacement rate, collision-loss rate, and the conditional mean, median, and 95th percentile event-message MAC access delay.

To examine whether CBR and BFR provide redundant contention information, an additional association analysis was performed using the frozen MIRA-EDCA outputs from the primary evaluation. The analysis uses all 900 MIRA-EDCA realizations corresponding to the 30 density–traffic operating points and 30 Monte Carlo realizations per operating point. Pearson correlation was used to quantify linear association, while Spearman rank correlation was additionally calculated to characterize monotonic dependence. Because both indicators may respond to common changes in vehicle population and event-message load, a pooled correlation alone may overstate their direct dependence. Therefore, a within-operating-point analysis was also performed by subtracting the corresponding (*N*, EventScale) operating-point mean from both CBR and BFR before recomputing the association. This residual analysis evaluates whether the two indicators remain strongly coupled after the common density–traffic operating condition has been controlled. No MIRA-EDCA parameter was changed as part of this post-processing analysis.

### 2.6. Ablation and Sensitivity Evaluation

A separate ablation experiment is used to identify the contribution of the contention indicators used by MIRA.

Four configurations are considered: Standard EDCA, Density-only, Joint-Load, and MIRA-EDCA.

For consistency with the existing ablation labels, the term Density-only is retained as shorthand for the variant using only the normalized local contender indicator,

*Ψ_i_* = *ρ_N_* (56)

it does not denote a physical vehicle-density quantity expressed in vehicles per unit distance.

The Joint-Load variant uses

*Ψ_i_* = *ρ_N_C* (57)

whereas complete MIRA uses,

*Ψ_i_* = *ρ_N_*(*C* + *F* − *CF*) (58)

All adaptive variants use the same raw-CW formulation, quantization levels, and one-level-per-epoch transition mechanism. Consequently, their differences arise from the information supplied by the contention-pressure indicator rather than from different CW update procedures.

The ablation experiment uses the same *N* = 20:20:200, *EventScale* = {1, 3, 5}, 30 Monte Carlo realizations, 15 s measurement interval, and main seed family beginning at 710,000.

To determine whether the numerical equivalence between the Joint-Load variant and Standard EDCA is a structural property of the Density + CBR formulation or a consequence of the adopted CW quantization, an additional quantization-sensitivity experiment was performed. The frozen baseline quantization was retained as the primary configuration, with event-traffic CW state-count levels {4, 8, 16, 32, 64, 128} and periodic-traffic levels {8, 16, 32, 64, 128}. A separate refined quantization was then evaluated using event-traffic levels {4, 6, 8, 16, 32, 64, 128} and periodic-traffic levels {8, 12, 16, 32, 64, 128}.

The refinement changes only the first quantization interval. Under the baseline event-traffic configuration, the raw CW request $W_{\mathrm{raw},E}=\frac{4}{1-\Psi +\varepsilon }$ must exceed the midpoint between the first two admissible levels, 4 and 8, for the nearest quantized target to leave the baseline state. Neglecting the infinitesimal ε in the threshold calculation, this requires $\frac{4}{1-\Psi }>6$, and therefore Ψ > 1/3. With the refined event-traffic levels, the first transition is instead determined by the midpoint between 4 and 6, so that $\frac{4}{1-\Psi }>5$, which gives Ψ > 0.20.

The same first-transition thresholds apply to the periodic-traffic levels because the baseline pair 8 → 16 has midpoint 12, whereas the refined pair 8 → 12 has midpoint 10. Thus, the sensitivity experiment reduces the first quantization threshold from approximately 0.333 to 0.20 without changing the Joint-Load or MIRA contention-pressure expressions.

Only the quantization level sets were changed in this sensitivity experiment. The contender-normalization parameter *W_ref_* = 8, 100 ms adaptation epoch, traffic-generation process, AIFS settings, message-validity rules, one-level-per-epoch transition constraint, 15 s measurement interval, 30 Monte Carlo realizations per operating point, and the matched seed family were retained. The baseline results were reused from the frozen ablation evaluation, whereas Joint-Load and complete MIRA-EDCA were re-evaluated under the refined quantization using the same operating points and matched random seeds. The refined configuration is used only as a sensitivity test and does not replace or retune the frozen primary controller.

Sensitivity is evaluated separately using a dedicated seed family beginning at 810,000.

The sensitivity grid contains *N* ∈ {100, 160, 200}, and EventScale ∈ {1, 5}, with 20 Monte Carlo realizations per configuration.

The first sensitivity experiment evaluates *W_ref_* ∈ {4, 8, 16}.

The baseline value remains *W_ref_* = 8; the alternative values are used only to assess sensitivity and are not used to replace the frozen primary configuration.

The second sensitivity experiment examines sensitivity to static error in the nominal local contender-count input. The contender-count proxy derived from the deterministic effective local population defined in Equations (1)–(3) is perturbed according to

(59) $${\hat{N}}_{\Delta }=\left(1+\Delta \right)\hat{N}$$

where the relative estimation error is varied over {−20%, 0, +20%}. The perturbation is applied as a fixed multiplicative bias to the nominal contender-count input for each realization. This experiment therefore evaluates whether moderate underestimation or overestimation of the deterministic local contention-population proxy materially changes the MIRA-EDCA operating point.

Importantly, this sensitivity test does not introduce explicit vehicle coordinates, time-varying neighborhood membership, neighbor-table aging, delayed neighbor information, or stochastic time-dependent estimation noise. It should therefore be interpreted as a bounded sensitivity analysis of the nominal contender-count input rather than as validation of a practical dynamic neighbor-estimation process.

A third sensitivity experiment examines adaptation-epoch duration using *T_epoch_* ∈ {50, 100, 200} ms. For this clean sensitivity experiment, the physical busy-period accounting grid is maintained at 100 ms for all three variants. Only the MIRA controller observation and update interval is changed. This separation ensures that changing *T_epoch_* does not simultaneously alter the model’s physical busy-period clipping boundary. The 100 ms clean configuration was compared with the previously frozen sensitivity baseline. The maximum absolute difference across the checked metrics was below 10^−12^, confirming numerical consistency between the baseline implementations.

A fourth sensitivity experiment examines the influence of the measurement duration on the reported performance metrics. The measurement interval is varied as *T_meas_* ∈ {15, 30, 60} s, while all other model parameters and MIRA-EDCA settings are kept unchanged. The experiment uses the same sensitivity grid, *N* = {100, 160, 200} and EventScale = {1, 5}, with 20 Monte Carlo realizations for each configuration. The 15 s interval corresponds to the frozen primary setting, whereas the 30 s and 60 s intervals are used only to assess sensitivity to the finite measurement horizon and are not used to retune the proposed method.

The sensitivity experiments are used only to characterize the response of the frozen MIRA-EDCA controller to the selected parameter variations, static contender-count bias, adaptation-epoch duration, and finite measurement horizon. They are not intended to establish robustness to time-varying measurement uncertainty or practical deployment conditions. In particular, the present experiments do not model delayed or stale neighbor-table information, temporally correlated contender-count errors, burst-dependent CBR noise, or small-sample variability of the BFR estimate. Consequently, the reported sensitivity results should not be interpreted as evidence of deployability under dynamic vehicular measurement conditions.

## 3. Results

The performance of the proposed MIRA-EDCA method was evaluated under the settings described in Section 2. The results are presented in terms of collision probability, transmitter-side event-message successful broadcast ratio (SBR), conditional event-message MAC access delay, event-message terminal outcomes, and contention-window behavior. The comparative analysis is followed by ablation and sensitivity evaluations of the proposed multi-indicator adaptation mechanism.

### 3.1. Collision Probability Under Increasing Vehicle Population

**Figure 2:**
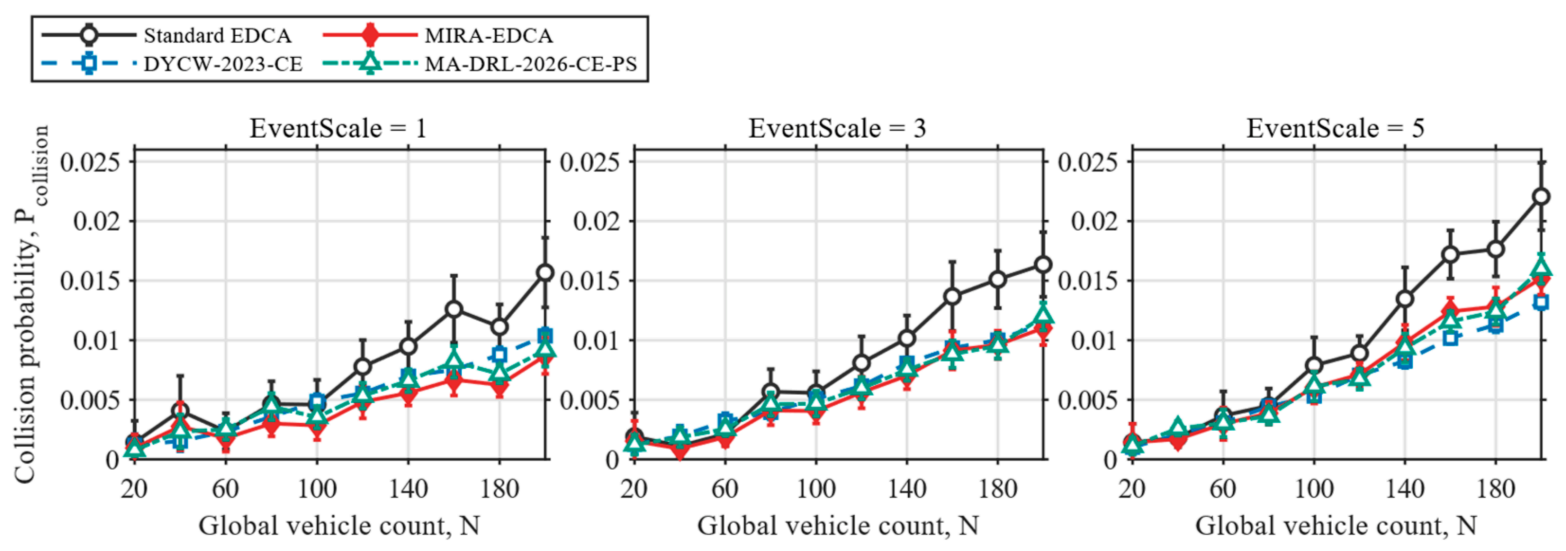
Collision probability versus global vehicle population for Standard EDCA, DYCW-2023-CE, MIRA-EDCA, and MA-DRL-2026-CE-PS under EventScale = 1, 3, and 5. Error bars indicate 95% confidence intervals over 30 Monte Carlo realizations.

Figure 2 presents the collision probability of Standard EDCA, DYCW-2023-CE, MIRA-EDCA, and MA-DRL-2026-CE-PS as a function of the global vehicle population. The comparison is performed for three event-traffic conditions, EventScale = 1, 3, and 5. For each operating point, the reported value is averaged over 30 Monte Carlo realizations, and the error bars indicate the corresponding 95% confidence intervals.

As shown in Figure 2, the collision probability increases with the number of vehicles for all considered methods. This increase becomes more pronounced as the event-message load changes from EventScale = 1 to EventScale = 5. Standard EDCA exhibits the highest collision probability over most medium- and high-density operating points, whereas the three adaptive methods maintain lower collision levels as the contention population increases.

Across the 30 operating points of the primary density–traffic grid, the mean collision probability is 0.008407 for Standard EDCA, 0.005972 for DYCW-2023-CE, 0.005724 for MIRA-EDCA, and 0.006049 for MA-DRL-2026-CE-PS. Relative to Standard EDCA, MIRA-EDCA reduces the overall mean collision probability by 31.92%. The difference becomes more evident in the high-density region. For *N* ≥ 100, the mean collision probability decreases from 0.012080 with Standard EDCA to 0.008046 with MIRA-EDCA, corresponding to a 33.40% reduction. The corresponding mean values for DYCW-2023-CE and MA-DRL-2026-CE-PS are 0.008305 and 0.008375, respectively. At *N* = 200, after averaging the three event-load conditions, the collision probabilities are 0.018038, 0.011667, 0.011642, and 0.012389 for Standard EDCA, DYCW-2023-CE, MIRA-EDCA, and MA-DRL-2026-CE-PS, respectively. Thus, MIRA-EDCA achieves a 35.46% reduction relative to Standard EDCA at the maximum evaluated vehicle population. At this density, the collision probabilities of MIRA-EDCA and DYCW-2023-CE are very close, indicating that collision probability alone does not fully characterize the behavior of the compared adaptive mechanisms. The paired analysis confirms the consistency of the reduction relative to Standard EDCA. Under the two-sided paired Student’s *t*-test with Holm–Bonferroni correction across the 30 operating points, the MIRA-EDCA collision-probability difference relative to Standard EDCA is statistically significant at 20 of the 30 operating points and at all 18 high-density operating points with *N* ≥ 100. The relative behavior of MIRA-EDCA, DYCW-2023-CE, and MA-DRL-2026-CE-PS is further examined using the SBR, access-delay, and contention-window results in the following subsections.

### 3.2. Event-Message Successful Broadcast Ratio

**Figure 3:**
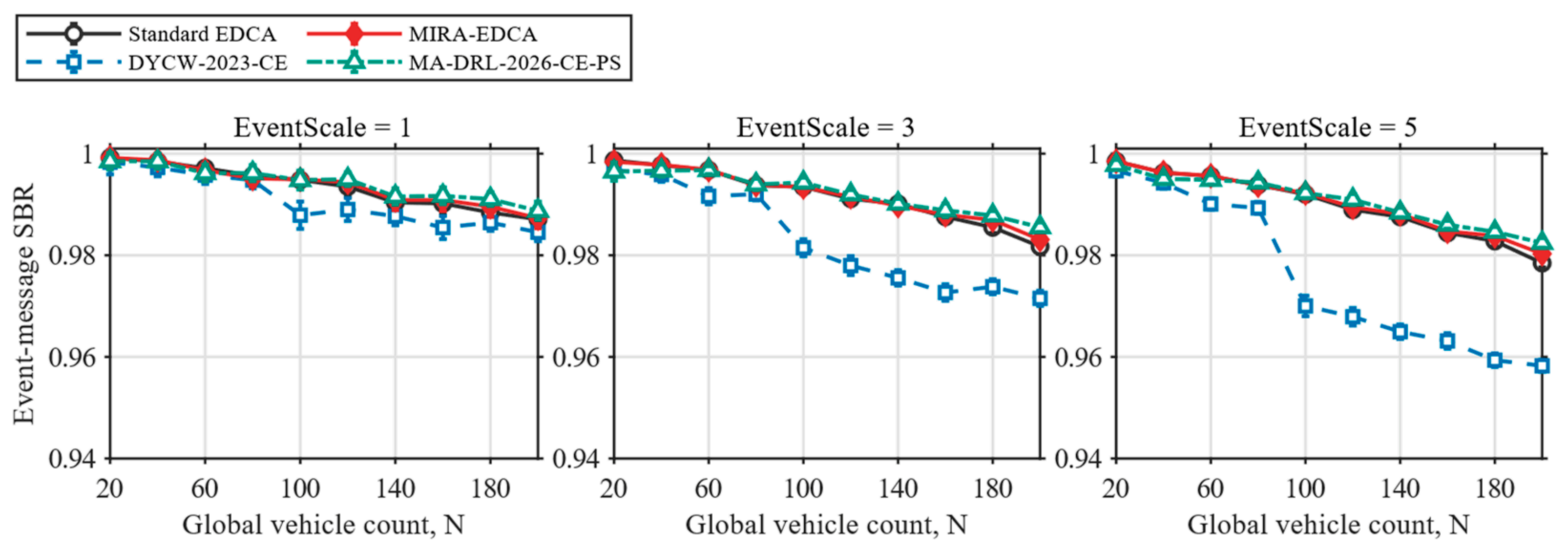
Event-message Successful Broadcast Ratio versus global vehicle population for Standard EDCA, DYCW-2023-CE, MIRA-EDCA, and MA-DRL-2026-CE-PS under EventScale = 1, 3, and 5. Error bars indicate 95% confidence intervals over 30 Monte Carlo realizations.

Figure 3 presents the event-message Successful Broadcast Ratio (SBR) as a function of the global vehicle population for Standard EDCA, DYCW-2023-CE, MIRA-EDCA, and MA-DRL-2026-CE-PS. The three panels correspond to EventScale = 1, 3, and 5. SBR is evaluated as the transmitter-side ratio of successfully completed event-message broadcasts to generated event messages, as defined in Section 2.5. Each operating point represents the mean of 30 Monte Carlo realizations, with error bars indicating the corresponding 95% confidence intervals.

As shown in Figure 3, the event-message SBR decreases gradually as the number of vehicles increases. The reduction becomes more pronounced under higher event-message loads, particularly for EventScale = 3 and EventScale = 5. Standard EDCA, MIRA-EDCA, and MA-DRL-2026-CE-PS retain comparatively similar SBR values over much of the evaluated range, whereas DYCW-2023-CE exhibits a more pronounced decrease at medium and high vehicle populations.

Across the 30 operating points of the primary density–traffic grid, the mean transmitter-side event-message SBR is 0.991656 for Standard EDCA, 0.983015 for DYCW-2023-CE, 0.992009 for MIRA-EDCA, and 0.992378 for MA-DRL-2026-CE-PS. Relative to Standard EDCA, the mean SBR difference for MIRA-EDCA is only approximately +0.035 percentage points, whereas the difference relative to the implemented DYCW-2023-CE comparator is approximately +0.899 percentage points. MA-DRL-2026-CE-PS differs from MIRA-EDCA by approximately +0.037 percentage points.

For N ≥ 100, the mean transmitter-side SBR of MIRA-EDCA differs from Standard EDCA by approximately +0.063 percentage points and from DYCW-2023-CE by approximately +1.34 percentage points. At *N* = 200, after averaging the three event-load conditions, the corresponding SBR values are 0.982414 for Standard EDCA, 0.971434 for DYCW-2023-CE, 0.983561 for MIRA-EDCA, and 0.985566 for MA-DRL-2026-CE-PS.

Under the most demanding evaluated operating point, *N* = 200 and EventScale = 5, the transmitter-side event-message SBR is 0.978460 for Standard EDCA, 0.958247 for DYCW-2023-CE, 0.980289 for MIRA-EDCA, and 0.982427 for MA-DRL-2026-CE-PS. The corresponding differences are approximately +0.183 percentage points between MIRA-EDCA and Standard EDCA, +2.20 percentage points between MIRA-EDCA and DYCW-2023-CE, and +0.214 percentage points between MA-DRL-2026-CE-PS and MIRA-EDCA.

After applying the two-sided paired Student’s *t*-test with Holm–Bonferroni correction, the small transmitter-side SBR difference between MIRA-EDCA and Standard EDCA is statistically significant at only 1 of the 30 operating points. The MIRA-EDCA versus DYCW-2023-CE SBR difference remains statistically significant at 19 of the 30 operating points, whereas no MIRA-EDCA versus MA-DRL-2026-CE-PS SBR difference remains statistically significant after multiplicity correction. Therefore, the slightly higher overall mean SBR of the learning-based comparator should not be interpreted as a systematic statistically supported advantage over MIRA-EDCA. These SBR comparisons characterize transmitter-side completion of the modeled contention and broadcast-access process only and should not be interpreted as receiver-side packet delivery ratio or end-to-end reliability. The corresponding MAC access-delay and contention-window results are examined in Section 3.3 and Section 3.4.

### 3.3. Event-Message MAC Access Delay

**Figure 4:**
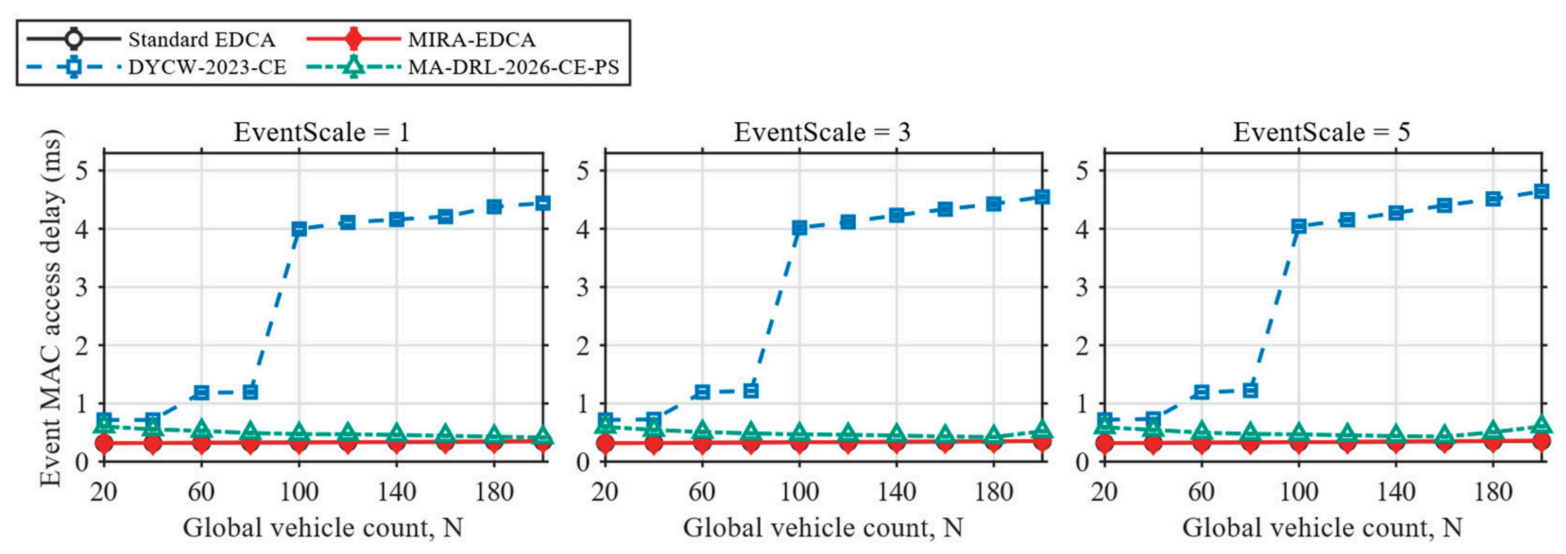
Mean event-message MAC access delay, conditional on successful transmission, versus global vehicle population for Standard EDCA, DYCW-2023-CE, MIRA-EDCA, and MA-DRL-2026-CE-PS under EventScale = 1, 3, and 5.

Figure 4 shows the conditional mean MAC access delay of successfully transmitted event messages for Standard EDCA, DYCW-2023-CE, MIRA-EDCA, and MA-DRL-2026-CE-PS. The results are presented for EventScale = 1, 3, and 5 as the global vehicle population increases from *N* = 20 to *N* = 200. Each point represents the mean over 30 Monte Carlo realizations.

As shown in Figure 4, Standard EDCA and MIRA-EDCA maintain comparatively low event-message access delay over the complete vehicle-density range. MA-DRL-2026-CE-PS operates at a higher delay level, whereas DYCW-2023-CE exhibits the largest increase as the number of vehicles grows.

Across the 30 operating points of the primary density–traffic grid, the mean event-message MAC access delay is 0.3341 ms for Standard EDCA, 2.9506 ms for DYCW-2023-CE, 0.3363 ms for MIRA-EDCA, and 0.4921 ms for MA-DRL-2026-CE-PS. Relative to Standard EDCA, the mean delay of MIRA-EDCA increases by only 0.66%. In contrast, within the implemented proxy comparison under matched evaluation assumptions, the mean event-message MAC access delay of MIRA-EDCA is approximately 88.6% lower than that of DYCW-2023-CE and 31.7% lower than that of MA-DRL-2026-CE-PS. The same ordering is maintained in the high-density region. For *N* ≥ 100, the mean event-message access delays are 0.3418 ms, 4.2774 ms, 0.3452 ms, and 0.4634 ms for Standard EDCA, DYCW-2023-CE, MIRA-EDCA, and MA-DRL-2026-CE-PS, respectively. For the same implemented proxy comparison in the high-density subset, the corresponding delay differences are approximately −91.9% relative to DYCW-2023-CE and −25.5% relative to MA-DRL-2026-CE-PS. At the maximum evaluated population *N* = 200, after averaging over the three event-load conditions, Standard EDCA achieves a mean event delay of 0.3514 ms. The corresponding value for MIRA-EDCA is 0.3573 ms, compared with 4.5437 ms for DYCW-2023-CE and 0.5141 ms for MA-DRL-2026-CE-PS. Thus, the event-message delay of MIRA-EDCA remains close to that of the fixed Standard EDCA configuration even at the highest evaluated vehicle population. The difference is particularly visible under the most demanding condition, *N* = 200, EventScale = 5. At this operating point, the mean event-message access delay is 0.3561 ms for Standard EDCA, 0.3634 ms for MIRA-EDCA, approximately 4.64 ms for DYCW-2023-CE, and 0.6054 ms for MA-DRL-2026-CE-PS. At this operating point, MIRA-EDCA maintains sub-millisecond event-message access delay, while the implemented DYCW-2023-CE and MA-DRL-2026-CE-PS common-environment comparators exhibit higher mean access delays. The paired statistical analysis confirms that these differences are systematic. The Holm-corrected paired analysis confirms that the small increase in the conditional mean event-message MAC access delay of MIRA-EDCA relative to Standard EDCA is statistically significant at 25 of the 30 operating points and at all 18 high-density operating points with *N* ≥ 100. Although the difference is statistically detectable over much of the evaluated grid, its absolute magnitude remains small, as reflected by the overall mean increase of only 0.66%.

For the implemented adaptive proxy comparators, the latency separation is substantially larger. MIRA-EDCA has a lower conditional mean event-message MAC access delay than DYCW-2023-CE at all 30 operating points, with the Holm-adjusted paired difference statistically significant at all 30 points. The corresponding conditional mean delay is also lower than that of MA-DRL-2026-CE-PS at all 30 operating points, again with Holm-adjusted statistical significance at all 30 points.

At the most demanding evaluated operating point, *N* = 200 and EventScale = 5, the paired conditional-mean delay difference is approximately +0.00731 ms for MIRA-EDCA relative to Standard EDCA and −0.2420 ms relative to MA-DRL-2026-CE-PS. Both differences remain statistically significant after Holm–Bonferroni correction. The first difference is small in absolute magnitude, whereas the separation from the learning-based proxy is substantially larger.

These results indicate that the delay increase in MIRA-EDCA relative to Standard EDCA remains numerically small, whereas the observed delay separation from the implemented learning-based comparator is substantially larger under the evaluated conditions.

Taken together with the collision and SBR results in Section 3.1 and Section 3.2, Figure 4 shows that the compared adaptive methods achieve different access-delay levels despite operating at relatively similar collision probabilities in several high-density conditions. The corresponding contention-window behavior is examined in the next subsection.

#### Event-Message Terminal Outcomes and Conditional Delay Distribution

Because the mean MAC access delay is defined only for successfully transmitted event messages, an additional outcome analysis was performed to quantify the event messages that did not complete successfully. The diagnostic evaluation retained the frozen controller parameters, traffic realizations, and matched seed structure of the primary comparison; the additional instrumentation reproduced the original primary metrics with a maximum absolute numerical difference of 4.97 × 10^−14^, confirming that the event-outcome accounting did not alter the evaluated contention-control behavior.

Across the 30 operating points, the mean incomplete-event rates were 0.8344% for Standard EDCA, 1.6985% for DYCW-2023-CE, 0.7991% for MIRA-EDCA, and 0.7622% for MA-DRL-2026-CE-PS. No event-message expiration and no residual pending event message were observed for any of the four methods under the evaluated 50 ms validity and drain rules. The corresponding event-replacement rates were 0.0285%, 1.1013%, 0.0292%, and 0.0900%, respectively, while the collision-loss rates were 0.8059%, 0.5972%, 0.7699%, and 0.6722%. Thus, the incomplete-event rate is not interpreted through latency truncation; instead, unsuccessful messages are accounted for explicitly through their terminal outcomes. In particular, the higher incomplete rate of the implemented DYCW-2023-CE proxy is dominated by event replacement rather than deadline expiration.

The conditional delay distribution provides additional context beyond the previously reported mean. Across the complete density–traffic grid, the mean/median/95th-percentile event-message MAC access delays were 0.3341/0.3181/0.4795 ms for Standard EDCA, 2.9506/2.9246/5.4286 ms for DYCW-2023-CE, 0.3363/0.3182/0.4837 ms for MIRA-EDCA, and 0.4921/0.3657/1.0293 ms for MA-DRL-2026-CE-PS. Consequently, MIRA-EDCA remains close to Standard EDCA not only in conditional mean delay but also in the center and upper tail of the successful-transmission delay distribution, whereas the two adaptive proxy implementations exhibit larger conditional-delay tails under the matched evaluation assumptions.

At the most demanding evaluated condition, *N* = 200 and EventScale = 5, the conditional 95th-percentile delay is approximately 0.5822 ms for Standard EDCA, 8.8056 ms for DYCW-2023-CE, 0.5947 ms for MIRA-EDCA, and 1.0940 ms for MA-DRL-2026-CE-PS. The corresponding incomplete-event rates are 2.1540%, 4.1753%, 1.9711%, and 1.7573%, respectively. These outcome rates should be interpreted jointly with the conditional delay statistics because the latter describe only the subset of event messages that complete successful transmissions.

### 3.4. Contention-Window Behavior

**Figure 5:**
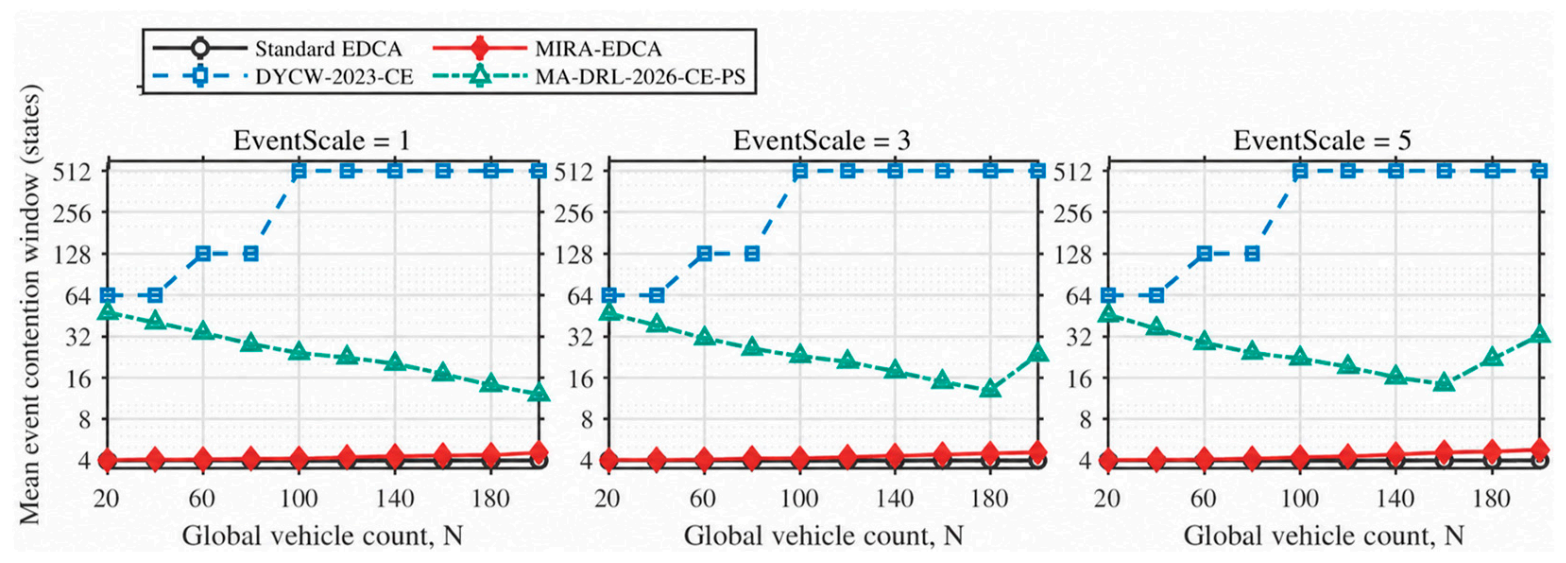
Mean active event-traffic CW state count *W_E_* versus global vehicle population for Standard EDCA, DYCW-2023-CE, MIRA-EDCA, and MA-DRL-2026-CE-PS under EventScale = 1,3, and 5. The vertical axis is shown on a logarithmic scale. All reported CW values denote state counts rather than conventional maximum backoff indices.

Figure 5 presents the mean active event-traffic CW state count WEW_E_ as a function of the global vehicle population. The results are shown for EventScale = 1,3, and 5. A logarithmic vertical scale is used because the compared methods operate over substantially different CW state-count ranges.

MIRA-EDCA remains close to the baseline CW state count over most of the evaluated operating range. Across the 30 operating points of the primary density–traffic grid, its mean active event CW state count is 4.269, compared with the fixed Standard EDCA state count *W_E_* = 4. In the high-density subset with *N* ≥ 100, the corresponding MIRA-EDCA mean state count increases only to approximately 4.40. At the maximum evaluated vehicle population *N* = 200, the mean active event CW state count, averaged across the three event-load conditions, is approximately 4.65.

DYCW-2023-CE exhibits a substantially different operating range. According to the density-to-CW rule used by this benchmark, the local contention population determines one of three discrete CW state counts. In the common evaluation environment, a global population of *N* = 20 corresponds to an effective local population of approximately seven vehicles and therefore activates *W_E_* = 64. For *N* = 60, the effective local population increases to approximately 19 vehicles, resulting in *W_E_* = 128. From approximately *N* = 100, the effective contender population exceeds 30 vehicles and the DYCW-2023-CE event CW state count increases to *W_E_* = 512. Consequently, the mean event CW state count of DYCW-2023-CE across the primary density–traffic grid is approximately 345.6, and its mean value over the *N* ≥ 100 subset is 512.

MA-DRL-2026-CE-PS also operates with CW state counts larger than those of Standard EDCA and MIRA-EDCA, although its values remain substantially below those selected by DYCW-2023-CE. Across the primary density–traffic grid, the mean event CW state count of the learning-based comparator is approximately 25.89. For *N* ≥ 100, its average value is approximately 19.38. The resulting ordering of the mean event CW state count is therefore Standard EDCA < MIRA-EDCA < MA-DRL-2026-CE-PS < DYCW-2023-CE.

The difference is substantial in numerical terms. The overall mean event CW state count of MIRA-EDCA is only about 6.7% larger than that of Standard EDCA, whereas MA-DRL-2026-CE-PS uses an average event CW state count approximately 6.5 times the Standard EDCA value. DYCW-2023-CE operates at an average event CW state count approximately 86 times the fixed Standard EDCA value over the primary density–traffic grid.

The effect of event-load intensity is also visible in the MIRA-EDCA and MA-DRL-2026-CE-PS curves. For MIRA-EDCA, the active event CW state count increases selectively as both vehicle density and event-message activity become more demanding, but it remains close to the lower admissible state-count levels. In contrast, the DYCW-2023-CE state count is primarily determined by the effective vehicle population and therefore remains at the same density-selected level across different EventScale conditions.

Taken together with Figure 2, Figure 3 and Figure 4, the CW results show that methods exhibiting similar collision probabilities can operate with substantially different contention-window state counts. In particular, at *N* = 200, MIRA-EDCA and DYCW-2023-CE achieve nearly identical average collision probabilities–approximately 0.01164 and 0.01167, respectively–while their mean active event CW state counts remain substantially different: approximately 4.65 for MIRA-EDCA and 512 for DYCW-2023-CE.

This difference is also reflected in the corresponding event-message access delay reported in Section 3.3. A direct comparison of the combined collision and delay operating points is therefore presented in the following subsection.

### 3.5. Collision–Access-Delay Operating Point

**Figure 6:**
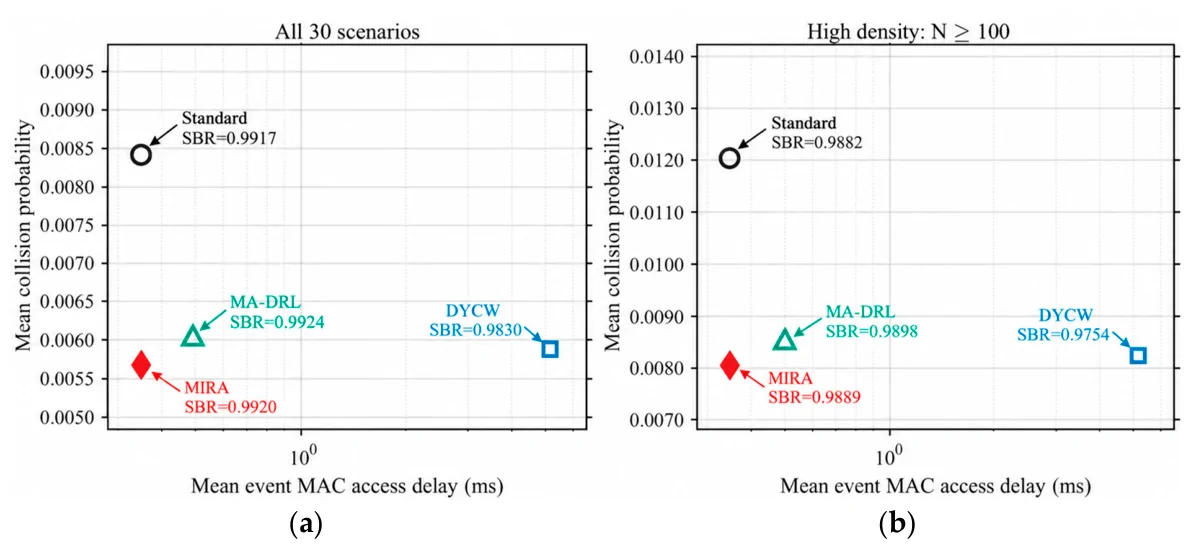
Collision–conditional-access-delay operating points of Standard EDCA, DYCW-2023-CE, MIRA-EDCA, and MA-DRL-2026-CE-PS: (**a**) mean results over the 30 operating points of the primary density–traffic grid; (**b**) mean results for the high-density subset *N* ≥ 100. The delay coordinate is conditional on successful event-message transmission. Marker annotations indicate the corresponding mean transmitter-side event-message Successful Broadcast Ratio.

Figure 6 summarizes the joint collision–access-delay behavior of the four evaluated contention-control methods. Rather than considering collision probability and MAC access delay independently, the figure places each method according to its mean collision probability and conditional mean event-message MAC access delay. The first panel represents the 30 operating points of the primary density–traffic grid, whereas the second panel considers only the high-density subset with N ≥ 100. The corresponding mean transmitter-side event-message SBR is provided as an additional annotation and is not used as a receiver-side reliability measure.

For the complete evaluation set, Standard EDCA operates at a mean collision probability of 0.008407 and a mean event-message MAC access delay of 0.3341 ms. Its corresponding mean event-message SBR is 0.991656. This operating point provides the lowest mean access delay among the four considered methods, but also the highest mean collision probability.

MIRA-EDCA shifts the operating point toward lower collision probability while remaining close to the Standard EDCA delay level. Its mean collision probability decreases to 0.005724, while the mean event-message MAC access delay is 0.3363 ms.

The corresponding mean event-message SBR is 0.992009. Thus, relative to Standard EDCA, the proposed method reduces the mean collision probability by 31.92%, while the mean event-message access delay increases by only 0.66%.

The implemented DYCW-2023-CE comparator reaches a mean collision probability of 0.005972, close to the MIRA-EDCA value of 0.005724. Under the same common-environment assumptions, its mean event-message access delay is 2.9506 ms and its mean SBR is 0.983015. Consequently, the two implementations occupy a similar collision-probability region but substantially different latency and transmitter-side successful-broadcast operating points.

The implemented learning-based MA-DRL-2026-CE-PS proxy produces a mean collision probability of 0.006049, a mean event-message MAC access delay of 0.4921 ms, and a mean SBR of 0.992378. Within this common-environment proxy comparison, its SBR is slightly higher than that of MIRA-EDCA, whereas MIRA-EDCA exhibits a 31.7% lower mean access delay and a slightly lower overall mean collision probability.

The relative positions become more distinct in the high-density subset shown in Figure 6b. For *N* ≥ 100, Standard EDCA produces a mean collision probability of 0.012080, a mean event-message access delay of 0.3418 ms, and an SBR of 0.988228. MIRA-EDCA reduces the collision probability to 0.008046 while maintaining an access delay of 0.3452 ms and an SBR of 0.988857.

This corresponds to a 33.40% reduction in collision probability relative to Standard EDCA, with the mean delay increase remaining below 1%.

For the same high-density conditions, DYCW-2023-CE reaches a mean collision probability of 0.008305, but its mean access delay increases to 4.2774 ms and its SBR decreases to 0.975425. MA-DRL-2026-CE-PS achieves a mean collision probability of 0.008375, an access delay of 0.4634 ms, and an SBR of 0.989786.

Accordingly, the high-density operating points can be summarized as follows in Table 4.

After Holm–Bonferroni correction, the paired comparison confirms that the latency separation between MIRA-EDCA and the implemented adaptive proxy comparators is consistent across the evaluation set. MIRA-EDCA provides a statistically significant lower event-message MAC access delay than DYCW-2023-CE at all 30 of 30 operating points and a statistically significant lower delay than MA-DRL-2026-CE-PS at all 30 of 30 operating points. For the high-density subset, the collision reduction of MIRA-EDCA relative to Standard EDCA is statistically significant at all 18 of 18 operating points.

Overall, Figure 6 shows that the four methods occupy different collision–access-delay operating regions. Standard EDCA provides the smallest MAC access delay but the largest collision probability, whereas DYCW-2023-CE provides strong collision suppression with substantially larger delay. MA-DRL-2026-CE-PS exhibits a transmitter-side SBR slightly above that of MIRA-EDCA, while operating at a higher mean MAC access delay. The MIRA-EDCA operating point remains close to Standard EDCA in terms of access delay while shifting toward the lower-collision region. The associated SBR values are reported only as transmitter-side completion metrics and are not interpreted as receiver-side reliability measurements.

### 3.6. Ablation Analysis of the MIRA Contention Indicators

**Figure 7:**
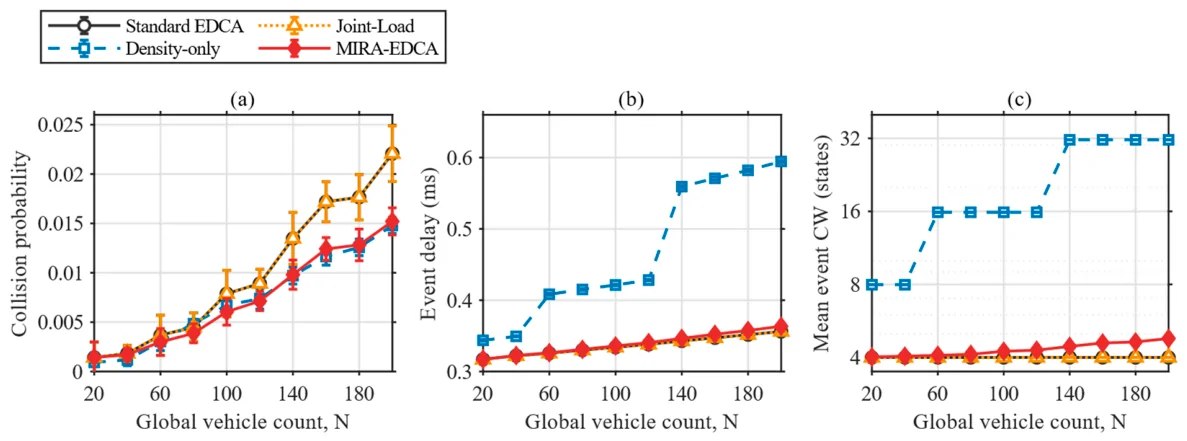
Ablation comparison of Standard EDCA, Density-only, Joint-Load, and complete MIRA-EDCA under EventScale = 5: (**a**) collision probability; (**b**) event-message MAC access delay; (**c**) mean active event CW state count. The adaptive variants use the same CW state-count quantization and one-level-per-epoch update procedure and differ only in the contention-pressure formulation.

Figure 7 presents the ablation results for Standard EDCA, the Density-only variant, the Joint-Load variant, and the complete MIRA-EDCA method. The experiment uses the same contention-window quantization and one-level-per-epoch update procedure for all adaptive variants; only the contention-pressure formulation is changed, as defined in Section 2.6. Figure 7 focuses on the EventScale = 5 condition to illustrate the behavior of the four configurations as vehicle density increases.

As shown in Figure 7, the Density-only variant reduces collision probability relative to Standard EDCA as vehicle population increases. However, this reduction is accompanied by a substantial increase in the active event CW state count and event-message MAC access delay. The Joint-Load variant, which combines the normalized local contender indicator and CBR, remains close to Standard EDCA over the evaluated range. In contrast, the complete MIRA-EDCA formulation achieves a lower collision probability while maintaining an event-message MAC access delay close to that of Standard EDCA.

Across the 30 operating points of the primary density–traffic grid, Standard EDCA produces a mean collision probability of 0.008407, whereas the Density-only configuration reduces this value to 0.006402. This corresponds to a collision reduction of approximately 23.85%. However, the mean event-message MAC access delay increases from 0.3341 ms to 0.4616 ms, representing an increase of approximately 38.15%. The mean active event CW state count simultaneously increases from *W_E_* = 4 to approximately 20.56.

The Joint-Load formulation produces an overall mean collision probability of 0.008407, a transmitter-side event-message SBR of 0.991656, and an event-message MAC access delay of 0.3341 ms. These values are numerically equivalent to the corresponding Standard EDCA results under the adopted numerical tolerance. The mean active event CW state count also remains at *WE* = 4.

The complete MIRA-EDCA method produces a mean collision probability of 0.005724 while maintaining a mean event-message MAC access delay of 0.33634 ms. Its mean active event CW state count is approximately 4.269. Relative to the Joint-Load variant, the complete MIRA-EDCA formulation reduces the mean collision probability by approximately 31.92%, while increasing the mean event-message MAC access delay by only 0.66%.

Accordingly, the overall ablation results can be summarized in Table 5.

The differences are also visible at the maximum evaluated vehicle population. At *N* = 200, after averaging over the three event-load conditions, Standard EDCA produces a mean collision probability of 0.018038 and an event-message MAC access delay of 0.3514 ms. The Density-only variant reduces the collision probability to 0.012720, but its mean delay increases to 0.5810 ms and its mean active event CW state count reaches approximately 31.55.

At the same vehicle population, the Joint-Load variant remains numerically equivalent to Standard EDCA, with a mean collision probability of 0.018038, a mean event-message MAC access delay of 0.3514 ms, and an event CW state count of *WE* = 4. In comparison, complete MIRA-EDCA reduces the collision probability to 0.011642 while maintaining an event-message MAC access delay of 0.3573 ms and a mean active event CW state count of approximately 4.65.

The transmitter-side event-message SBR at *N* = 200 is 0.982414 for Standard EDCA, 0.985973 for Density-only, 0.982414 for Joint-Load, and 0.983561 for MIRA-EDCA. Thus, the Density-only configuration produces the highest transmitter-side SBR among the ablation variants at this operating point, but it does so together with a substantially larger event CW state count and higher MAC access delay.

These results demonstrate that the three ablation configurations lead to clearly different operating points. Density-only adaptation provides effective collision suppression but operates with considerably larger event CW state counts. The Density + CBR Joint-Load formulation produces little observable deviation from the fixed Standard EDCA configuration under the adopted quantized state-count update mechanism. The complete Density + CBR + BFR formulation achieves the lowest overall mean collision probability among the four configurations while keeping the event-message MAC access delay close to the Standard EDCA level.

#### Quantization-Sensitivity Check of the Joint-Load Ablation

The numerical equivalence between the baseline Joint-Load variant and Standard EDCA was further examined using the quantization-sensitivity experiment defined in Section 2.6. Under the frozen baseline quantization, Joint-Load exactly reproduced the Standard EDCA aggregate operating point across the 30 density–traffic conditions: the mean collision probability was 0.008407, the mean transmitter-side event-message SBR was 0.991656, the mean event-message MAC access delay was 0.33412 ms, and the mean active event CW state count remained at 4.000.

When only the first CW quantization interval was refined so that the first contention-pressure transition threshold decreased from approximately *Ψ* = 0.333 to *Ψ* = 0.20, the Joint-Load controller no longer remained numerically identical to Standard EDCA. Its overall mean active event CW state count increased from 4.000 to 4.032, and *W_E_* > 4 occurred at 5 of the 30 evaluated operating points. These transitions occurred under the higher-load conditions (EventScale, *N*) = (3, 180), (3, 200), (5, 160), (5, 180), and (5, 200).

Across the full 30-point grid, the refined Joint-Load configuration produced a mean collision probability of 0.008364, a mean transmitter-side event-message SBR of 0.991702, and a mean event-message MAC access delay of 0.33443 ms. Relative to Standard EDCA, this corresponds to an approximately 0.51% reduction in mean collision probability with an approximately 0.09% increase in mean event-message MAC access delay. Thus, the exact baseline equivalence of Joint-Load to Standard EDCA is not a structural property of the Density + CBR formulation itself; rather, it results from the interaction between the resulting contention-pressure values and the adopted discrete CW quantization boundaries.

The complete MIRA formulation was substantially less dependent on this first quantization boundary. Under the baseline quantization, MIRA-EDCA produced a mean collision probability of 0.005724, a mean event-message MAC access delay of 0.33634 ms, and a mean active event CW state count of 4.269. Under the refined quantization, the corresponding values were 0.005762, 0.33629 ms, and 4.263, respectively. MIRA-EDCA therefore continued to leave the baseline event CW state at all 30 operating points under both quantization structures, while its aggregate collision–delay operating point changed only slightly.

These results refine the interpretation of the original ablation study. BFR should not be interpreted as universally necessary for any Density + CBR-based CW adaptation, because Joint-Load can become active when the quantization boundary is lowered. Rather, under the frozen baseline controller, BFR provides additional node-level contention information that raises the bounded contention-pressure state sufficiently to produce more persistent and widespread CW adaptation without requiring a finer quantization grid. The quantitative results of the quantization-sensitivity analysis are summarized in Table 6.

Taken together, the primary ablation and the quantization-sensitivity experiment show that the baseline inactivity of the Joint-Load formulation is caused by its interaction with the adopted discrete CW thresholds rather than by an inherent inability of Density + CBR information to drive CW adaptation. The complete MIRA formulation nevertheless produces substantially more widespread adaptation and a markedly different collision–delay operating point under both quantization structures. The contribution of the complete multi-indicator formulation is further interpreted in Section 4, while sensitivity of the frozen primary MIRA-EDCA configuration to other controller parameters is examined next.

### 3.7. Sensitivity Analysis

The sensitivity of the frozen MIRA-EDCA configuration was evaluated separately from the primary comparison. The analysis characterizes the response to variations in the reference parameter, static error in the estimated local contender population, adaptation-epoch duration, and measurement horizon. These experiments quantify parameter dependence under the specified controlled conditions and are not intended as validation of robustness to time-dependent measurement errors or practical deployment uncertainty.

#### 3.7.1. Sensitivity to *W_ref_* and Static Contender-Count Input Bias

**Figure 8:**
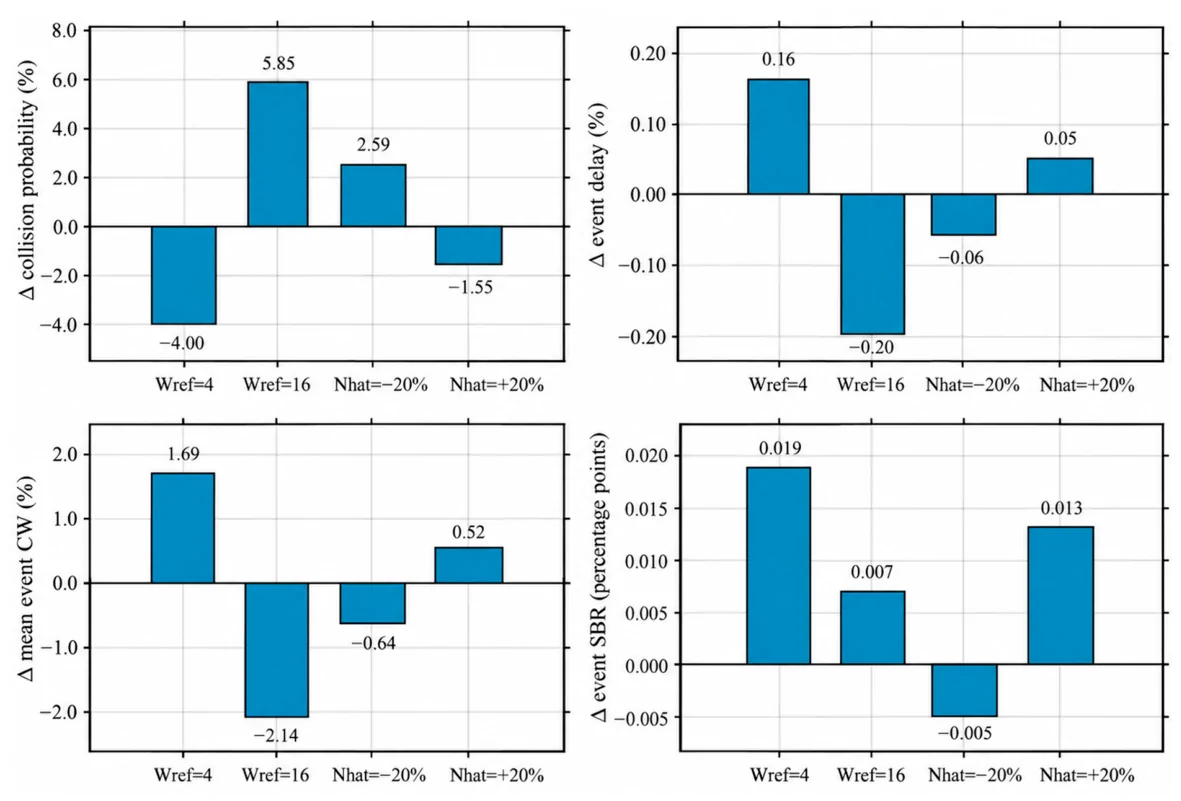
Sensitivity of MIRA-EDCA to variations in the reference contender-count parameter *W_ref_* and to static bias in the nominal contender-count input. Results are averaged over *N* = {100, 160, 200}, EventScale = {1, 5}, and 20 Monte Carlo realizations per configuration.

Figure 8 presents the sensitivity of MIRA-EDCA to variations in the contender-count normalization parameter *W_ref_* and to a controlled static bias in the nominal local contender-count input. The frozen primary configuration uses *W_ref_* = 8 and the nominal deterministic contender-count proxy defined in Section 2.1. The ±20% cases therefore represent fixed multiplicative underestimation and overestimation of this nominal input rather than explicit time-varying neighbor-estimation errors.

For the baseline sensitivity configuration, the mean collision probability is 0.008705, the mean transmitter-side event-message SBR is 0.987480, and the mean event-message MAC access delay is 0.3464 ms. The corresponding mean active event CW state count is approximately 4.43.

Reducing the reference contender-count parameter decreases the mean collision probability to 0.008356, corresponding to a reduction of approximately 4.0% relative to the baseline sensitivity configuration. The mean event-message MAC access delay changes only slightly, from 0.3464 ms to 0.3470 ms, while the mean active event CW state count increases from approximately 4.43 to 4.50.

For *W_ref_* = 16, the mean collision probability increases to 0.009214, approximately 5.85% above the baseline value. The mean event-message delay decreases slightly to 0.3457 ms, while the mean event CW state count decreases to approximately 4.33. The corresponding SBR values remain close across all three *W_ref_* settings.

The controlled static contender-count perturbations also produce relatively limited changes in the reported MAC-layer metrics. When the nominal contender-count input is reduced by 20%, the mean collision probability becomes 0.008930, corresponding to an increase of approximately 2.59% relative to the unperturbed input, while the mean event-message MAC access delay changes from 0.3464 ms to 0.3462 ms. With a 20% positive bias, the mean collision probability decreases to 0.008570, approximately 1.55% below the nominal result, while the mean event-message MAC access delay becomes 0.3466 ms.

The transmitter-side event-message SBR also changes only slightly under these static input-bias conditions. A 20% negative bias changes the mean SBR from 0.987480 to 0.987432, whereas a 20% positive bias produces a mean SBR of 0.987611.

Accordingly, within the evaluated model, a fixed ±20% multiplicative bias in the nominal contender-count input does not produce a substantial displacement of the MIRA-EDCA operating point. This result should be interpreted only as sensitivity to a bounded static input perturbation. It does not demonstrate robustness to time-varying neighborhood membership, delayed or stale neighbor information, spatial clustering, or temporally correlated estimation errors, none of which are represented in this experiment. The corresponding *W_ref_* and static contender-count-bias results are summarized in Table 7.

#### 3.7.2. Adaptation-Epoch Sensitivity and the Stability–Responsiveness Trade-Off

**Figure 9:**
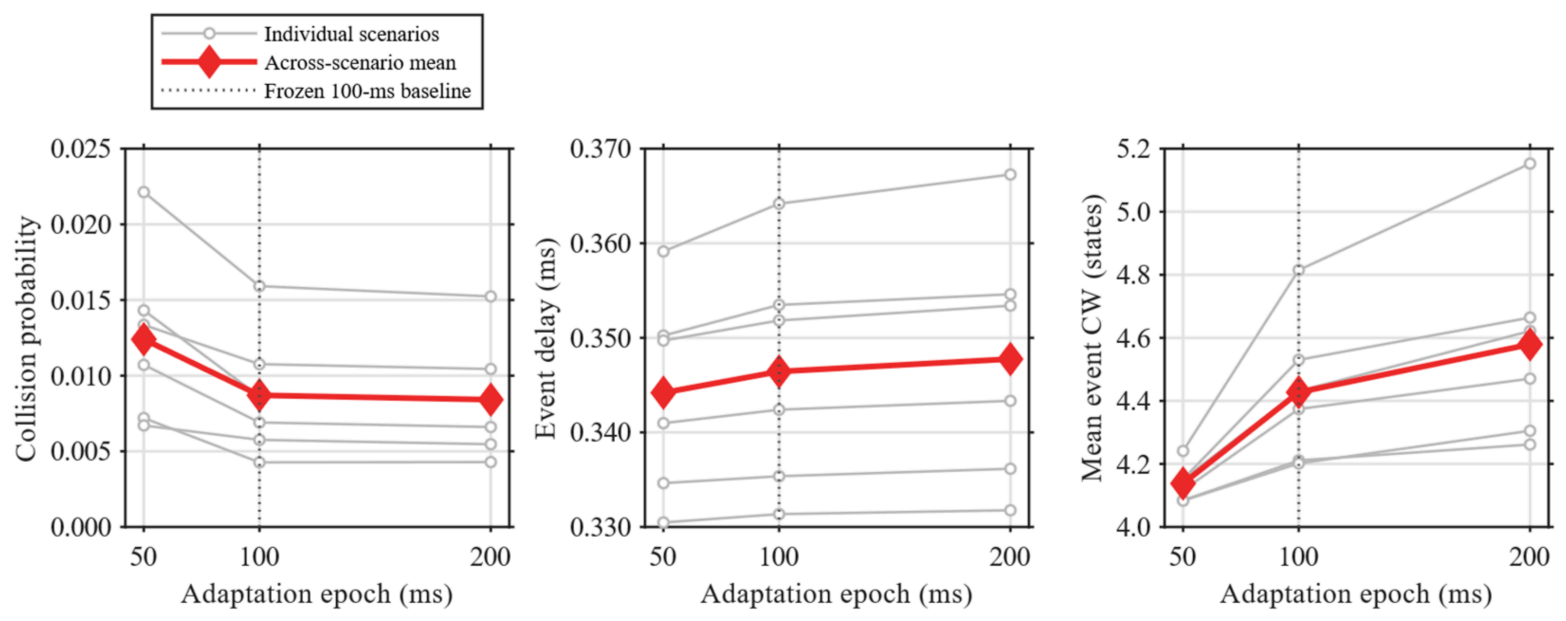
Sensitivity of MIRA-EDCA to the adaptation-epoch duration *T*_obs_. The physical busy-period accounting interval is fixed at 100 ms, while the MIRA adaptation interval is varied among 50, 100, and 200 ms. Results are averaged over *N* = {100, 160, 200}, EventScale = {1, 5}, and 20 Monte Carlo realizations per configuration. The comparison is intended to characterize the stability–responsiveness trade-off rather than to select an optimal adaptation interval.

Figure 9 evaluates the influence of the adaptation-epoch duration. Three values are considered: *T_obs_* ∈ {50, 100, 200} ms.

For this clean sensitivity experiment, the physical busy-period accounting interval is maintained at 100 ms for all three cases. Therefore, only the MIRA observation and update interval is varied, avoiding simultaneous modification of the underlying channel-busy accounting procedure.

The 100 ms configuration reproduces the previously frozen sensitivity baseline, with a mean collision probability of 0.008705, a mean transmitter-side event-message SBR of 0.987480, a mean event-message MAC access delay of 0.3464 ms, and the corresponding mean active event CW state count reported for the baseline sensitivity configuration. The maximum numerical difference between the clean 100 ms implementation and the previously frozen baseline is below 3.6 × 10^−14^, confirming numerical consistency between the two implementations.

Reducing the adaptation epoch to 50 ms increases the mean collision probability to approximately 0.012404, which is about 42.5% higher than the 100 ms result. At the same time, the mean event-message MAC access delay decreases slightly to approximately 0.3442 ms, and the mean active event CW state count decreases to approximately 4.14.

The paired comparison between the 50 ms and 100 ms configurations shows a statistically significant increase in collision probability in 5 of the 6 evaluated operating points. The reduction in event-message MAC access delay is statistically significant in all 6 of 6 operating points.

Increasing the adaptation epoch to 200 ms produces a mean collision probability of approximately 0.008413, which is about 3.36% lower than the 100 ms value. The corresponding mean event-message MAC access delay increases slightly to 0.3478 ms, and the mean active event CW state count increases to approximately 4.58.

The collision difference between the 200 ms and 100 ms configurations is statistically significant in only 1 of the 6 operating points, whereas the increase in event-message MAC access delay is statistically significant in 5 of the 6 operating points. The mean active event CW state count is higher for the 200 ms configuration in all six evaluated operating points.

These results indicate that the adaptation epoch is an important controller-design parameter because it determines a trade-off between responsiveness and temporal stability. A shorter epoch allows the controller to react more frequently to changes in the observed contention state, but it also makes the CW update more sensitive to short-term fluctuations in channel occupancy and backoff interruption. In the present experiment, this behavior is reflected by the 50 ms configuration, which slightly reduces event-message MAC access delay but produces a substantially higher collision probability than the 100 ms baseline. In contrast, a longer epoch provides greater temporal aggregation of the contention observations and produces a more persistent state-count response, but updates the controller less frequently. The 200 ms configuration therefore shifts the operating point toward a slightly larger active CW state count and MAC access delay while producing only a modest reduction in mean collision probability relative to 100 ms.

Accordingly, the sensitivity study should not be interpreted as an optimization search for the best adaptation interval. The 100 ms configuration represents the pre-specified and frozen baseline used in the primary evaluation. It occupies an intermediate control timescale between the more reactive 50 ms setting and the more persistent 200 ms setting. The 200 ms result does not establish a superior configuration because its collision reduction relative to 100 ms is small and statistically significant in only one of the six evaluated operating points, whereas its increase in event-message MAC access delay is statistically significant in five of the six operating points. The choice of 100 ms is therefore retained as a balanced baseline for the evaluated traffic model rather than claimed as a globally optimal epoch duration.

#### 3.7.3. Measurement-Duration Sensitivity

To examine whether the 15 s measurement horizon used in the primary evaluation materially affects the reported MAC-layer performance estimates, an additional duration-sensitivity experiment was conducted using measurement intervals of 15, 30, and 60 s. The frozen MIRA-EDCA configuration was retained without modification, with *W_ref_* = 8, a 100 ms adaptation epoch, and the nominal local-contender estimate. The experiment used *N* = {100, 160, 200}, EventScale = {1, 5}, and 20 Monte Carlo realizations for each operating point and measurement duration.

Table 8 summarizes the resulting mean collision probability, transmitter-side event-message SBR, event-message MAC access delay, and mean active event CW state count. The reported values are expressed as mean ± 95% confidence-interval half-width over the 20 Monte Carlo realizations after averaging the six sensitivity operating points within each realization. The 15 s configuration reproduces the previously reported sensitivity baseline, providing an internal consistency check for the measurement-duration sensitivity experiment.

As shown in Table 8, increasing the measurement interval from 15 to 60 s does not materially alter the principal MAC access-delay or contention-window behavior of the frozen MIRA-EDCA configuration. Relative to the 15 s reference, the 60 s configuration changes the mean event-message MAC access delay by only −0.023%, from 0.34644 to 0.34636 ms, while the mean active event CW state count changes by −0.25%, from 4.427 to 4.416. The corresponding mean collision probability is 6.90% lower, changing from 0.008705 to 0.008104, whereas the transmitter-side event-message SBR increases by approximately 0.092 percentage points, from 0.987480 to 0.988401.

The collision-probability values are not strictly monotonic with measurement duration, indicating that this metric retains greater finite-realization variability than the access-delay and contention-window metrics. Nevertheless, the paired 60 s versus 15 s comparison shows no statistically significant difference in collision probability at any of the six evaluated operating points. Likewise, no statistically significant difference in event-message MAC access delay is observed at any of the six operating points. The transmitter-side SBR and mean event-CW differences are statistically significant at only one of the six operating points each.

These results indicate that extending the measurement horizon to 60 s does not produce a systematic displacement of the MIRA-EDCA MAC-layer operating point over the evaluated sensitivity grid. Accordingly, the original 15 s interval is retained as the frozen primary measurement setting rather than replaced after inspection of the sensitivity results. The longer 30 s and 60 s realizations are used solely to quantify sensitivity to the finite observation horizon and not to retune the proposed contention-control mechanism.

Overall, the sensitivity experiments quantify how the frozen MIRA-EDCA controller responds to the specific perturbations examined in this study. Static ±20% changes in the nominal contender-count input produce relatively small changes in the aggregated MAC-layer metrics, whereas adaptation-epoch duration has a stronger influence, particularly for the 50 ms setting. Extending the measurement horizon from 15 to 60 s does not produce a statistically significant change in collision probability or event-message MAC access delay across the six evaluated sensitivity operating points.

These observations are limited to the tested static parameter variations and finite measurement horizons. They do not establish robustness to delayed or stale neighbor information, time-correlated contender-count errors, burst-dependent CBR fluctuations, small-sample BFR variability, or combinations of these effects. Therefore, the present sensitivity analysis should not be interpreted as evidence of practical deployability or as a general robustness validation of MIRA-EDCA.

### 3.8. Relationship Between Channel Busy Ratio and Backoff Freeze Ratio

The relationship between CBR and BFR was examined using the 900 frozen MIRA-EDCA realizations of the primary density–traffic evaluation. When all operating points are pooled, the two indicators exhibit a positive association, with Pearson *r* = 0.720 (95% CI: 0.687–0.750) and Spearman ρ = 0.767. The corresponding *R*^2^ = 0.518 indicates that approximately 51.8% of the pooled linear variation is shared. This result is expected because increasing vehicle population and event-message load can simultaneously increase aggregate channel occupancy and the frequency with which pending backoff processes are interrupted.

The pooled association, however, does not imply that CBR and BFR are interchangeable measurements. In the high-density subset *N* ≥ 100, the Pearson correlation decreases to *r* = 0.626 (95% CI: 0.571–0.674), with Spearman *ρ* = 0.608. When evaluated separately by event-load condition, the Pearson correlations are 0.576, 0.702, and 0.805 for EventScale = 1, 3, and 5, respectively. Thus, the apparent dependence becomes stronger as the analysis spans operating points with progressively different contention levels.

To separate this common operating-condition effect from within-condition dependence, both indicators were demeaned within each of the 30 (*N*, EventScale) operating points. The resulting within-operating-point association is weak: Pearson *r* = −0.159 (95% CI: −0.222 to −0.095), Spearman *ρ* = −0.162 and *R*^2^ = 0.025. Therefore, after controlling for the common density–traffic operating condition, only approximately 2.5% of the residual linear variation is shared between the two indicators.

The same pattern is visible when the 30 operating points are examined individually. The median within-operating-point Pearson correlation is approximately −0.097, with values ranging from −0.513 to 0.329. None of the 30 operating points exhibits ∣*r*∣ ≥ 0.7, and only three exhibit ∣*r*∣ ≥ 0.5. These results indicate that CBR and BFR are related through their common response to contention intensity but are not redundant observations of the same MAC state.

This distinction follows directly from their definitions. CBR measures the fraction of the observation interval during which the medium is occupied, irrespective of whether a particular node is actively progressing through a backoff process. BFR instead measures the fraction of relevant backoff opportunities during which countdown progression is interrupted by medium activity. Consequently, two realizations with similar aggregate CBR can exhibit different BFR values depending on the temporal overlap between busy periods and active pending backoff processes. The empirical correlation analysis therefore supports treating CBR and BFR as complementary rather than statistically independent contention indicators. The corresponding correlation statistics are summarized in Table 9.

## 4. Discussion

The results show that the main contribution of MIRA-EDCA lies in combining potential contender population, aggregate channel occupancy, and node-level backoff interruption while preserving EDCA-based traffic priority. Relative to fixed Standard EDCA, the proposed mechanism shifts the operating point toward substantially lower contention/access collision probability while keeping event-message MAC access delay close to the fixed-CW reference. Across the primary grid, the mean collision reduction was 31.92% with only a 0.66% increase in the conditional mean event-message MAC access delay among successfully transmitted event messages; the same trade-off remained evident in the high-density subset. Importantly, this behavior was achieved while the mean active event CW state count remained close to the Standard EDCA value, indicating that collision reduction did not require persistent expansion of the high-priority backoff space.

The implemented DYCW-2023-CE proxy illustrates why collision suppression should be interpreted jointly with conditional MAC access delay and event-message terminal outcomes. Across the primary grid, its mean collision probability is 0.005972, close to the MIRA-EDCA value of 0.005724, but its conditional mean, median, and 95th-percentile event-message MAC access delays are 2.9506, 2.9246, and 5.4286 ms, respectively, compared with 0.3363, 0.3182, and 0.4837 ms for MIRA-EDCA. The corresponding incomplete-event rate is 1.6985% for DYCW-2023-CE and 0.7991% for MIRA-EDCA. Importantly, no event-message expiration was observed for either method under the evaluated 50 ms validity rule. For DYCW-2023-CE, the incomplete-event rate consists primarily of a 1.1013% replacement rate and a 0.5972% collision-loss rate, whereas for MIRA-EDCA the corresponding rates are 0.0292% and 0.7699%. Thus, the lower transmitter-side SBR of the implemented DYCW proxy is associated primarily with increased replacement of pending event messages rather than deadline expiration. This behavior is consistent with the substantially larger contention-window state counts and longer pending intervals produced by the transferred density-to-CW rule. These observations apply only to the implemented DYCW-2023-CE proxy under the matched evaluation assumptions and should not be interpreted as an evaluation of the complete cooperative architecture in [17].

The MA-DRL-2026-CE-PS comparison provides a complementary reference. The frozen learning-based proxy has a slightly higher overall mean transmitter-side event-message SBR than MIRA-EDCA (0.992378 versus 0.992009), but none of the 30 operating-point SBR differences remains statistically significant after Holm–Bonferroni correction. Therefore, this small aggregate difference should not be interpreted as a systematic advantage of the learning-based proxy. In contrast, the conditional delay distributions remain clearly separated under the implemented proxy comparison: MIRA-EDCA reduces the conditional mean, median, and 95th-percentile event-message MAC access delay by approximately 31.7%, 13.0%, and 53.0%, respectively, relative to MA-DRL-2026-CE-PS. The paired differences for all three delay summaries remain statistically significant at all 30 operating points after Holm correction. MIRA-EDCA also operates with a substantially smaller mean active event CW state count. These results characterize only the implemented CW-control strategies under matched assumptions and do not establish general superiority of deterministic adaptation over reinforcement learning or over the complete method represented by [23].

The additional event-outcome analysis also clarifies the interpretation of the reported latency results. The mean, median, and 95th-percentile MAC access delays are conditional statistics computed only for event messages that successfully complete transmission; they are not unconditional latency measures over all generated event messages. Assigning an artificial deadline or truncated delay to unsuccessful messages would combine fundamentally different terminal outcomes into a single latency quantity. Instead, this study reports unsuccessful outcomes separately through the incomplete-transmission, collision-loss, replacement, expiration, and residual rates. Across the complete evaluation grid, the incomplete-event rates are 0.8344% for Standard EDCA, 1.6985% for DYCW-2023-CE, 0.7991% for MIRA-EDCA, and 0.7622% for MA-DRL-2026-CE-PS. No event-message expiration or residual pending message was observed for any method. Consequently, the conditional-delay results should be interpreted jointly with these terminal-outcome rates rather than as latency measurements for messages that did not complete transmission.

The percentage differences reported for DYCW-2023-CE and MA-DRL-2026-CE-PS should therefore be interpreted strictly within these common-environment proxy implementations. The omitted or adapted source-specific components may affect performance in either direction, and the present model does not provide evidence that their omission systematically benefits MIRA-EDCA. Consequently, these comparisons are used to characterize differences among the implemented CW-control strategies under matched assumptions rather than to rank the complete source protocols.

Under the implemented learning-based proxy, MIRA-EDCA achieves a slightly lower mean collision probability and a slightly lower transmitter-side SBR, while providing a substantially lower conditional mean event-message MAC access delay. The small SBR difference relative to MA-DRL-2026-CE-PS is not statistically significant at any of the 30 operating points after Holm–Bonferroni correction, whereas the conditional mean-delay difference remains statistically significant at all 30 operating points. These results indicate comparable transmitter-side completion behavior within the evaluated conditions, together with a lower conditional MAC access-delay operating point for MIRA-EDCA. They do not establish general superiority over learning-based methods.

The ablation results provide additional insight into the role of the three contention indicators used by MIRA. Density-only adaptation uses the normalized contender population directly as contention pressure. Since this quantity remains relatively large at medium and high vehicle populations even when instantaneous channel activity is moderate, the resulting CW response is comparatively aggressive. Across the primary density–traffic grid, the Density-only configuration reduced collision probability by 23.85% relative to Standard EDCA but increased the mean event-message MAC access delay by 38.15%, while its mean active event CW state count increased from the Standard EDCA value *WE* = 4 to approximately 20.56. Density information is therefore useful for identifying the potential contention population, but it does not by itself distinguish between vehicles that are present in the contention domain and vehicles that are actively imposing sustained MAC contention.

The baseline Joint-Load result requires a more specific interpretation. Under the frozen CW quantization used in the primary ablation, the Density + CBR Joint-Load formulation remained numerically equivalent to Standard EDCA because its contention-pressure values did not cross the first quantization boundary sufficiently often to change the active CW state. For the baseline event-CW levels {4, 8, 16, 32, 64, 128}, the first nearest-level transition requires approximately *Ψ* > 1/3. The Joint-Load formulation had a mean contention pressure of approximately 0.093 across the complete evaluation set, so the resulting raw CW requests generally remained mapped to the baseline event-CW state.

The additional quantization-sensitivity experiment shows that this equivalence is a parameter-coupling effect rather than a structural property of the Density + CBR formulation. When only the first quantization boundary was reduced from approximately *Ψ* = 0.333 to *Ψ* = 0.20, while retaining the same Joint-Load expression, traffic assumptions, observation epoch, contender normalization, and one-level transition rule, Joint-Load left the baseline event-CW state at 5 of the 30 operating points. Its mean active event CW state count increased from 4.000 to 4.032, while the mean collision probability changed from 0.008407 to 0.008364 and the mean event-message MAC access delay from 0.33412 to 0.33443 ms. Thus, Density + CBR information can drive CW adaptation when the admissible CW structure makes the corresponding raw request observable at the quantized control output.

Accordingly, the ablation study should not be interpreted as demonstrating that BFR is universally necessary for CW adaptation. Rather, the role of BFR in the present controller is to provide additional node-level information about interruption of the local backoff process, which complements the aggregate occupancy information represented by CBR. Under the frozen baseline quantization, this additional information increases the fused contention-pressure state sufficiently to produce CW adaptation across a much broader portion of the evaluated operating space. MIRA-EDCA left the baseline event-CW state at all 30 operating points under both the baseline and refined quantization structures, whereas Joint-Load did so at 0 of 30 points under the baseline structure and 5 of 30 points under the refined structure. At the same time, the aggregate MIRA operating point changed only slightly when the first quantization interval was refined: mean collision probability changed from 0.005724 to 0.005762 and mean event-message MAC access delay from 0.33634 to 0.33629 ms.

The direct CBR–BFR association analysis in Section 3.8 further clarifies why BFR can add information beyond aggregate channel occupancy without implying that it is universally required for CW adaptation. Although the two indicators are positively correlated when heterogeneous density–traffic operating points are pooled (*r* = 0.720), their within-operating-point correlation after removal of the common operating-condition means is weak (*r* = −0.159, *R*^2^ = 0.025). Thus, their pooled association is driven substantially by their common response to increasing contention intensity, whereas their residual behavior within a fixed operating condition remains largely non-redundant. This supports the interpretation of CBR and BFR as complementary MAC observations rather than as statistically independent or interchangeable measurements. Taken together, these results indicate that the contribution of BFR is best interpreted as improved observability and responsiveness of the quantized controller under the frozen design, rather than as a prerequisite for all possible Density + CBR contention-control formulations.

The sensitivity analysis characterizes the response of the MIRA-EDCA operating point to the tested static variations in the contender-count input and its normalization. A −20% contender-estimation bias changed the mean collision probability by approximately +2.59%, whereas a +20% bias changed it by approximately −1.55%, with only minor changes in event-message MAC access delay and transmitter-side SBR. Adaptation-epoch duration had a stronger influence: reducing the epoch from 100 ms to 50 ms increased the mean collision probability, whereas the 200 ms setting produced a more persistent but less frequently updated response. The 100 ms value is therefore retained as the frozen baseline rather than interpreted as a globally optimal setting.

Measurement-duration analysis further showed that extending the measurement horizon from 15 to 60 s did not systematically displace the principal MAC-layer operating point over the evaluated sensitivity grid; in particular, the 60 s versus 15 s comparison produced no statistically significant difference in collision probability or event-message MAC access delay at any of the six evaluated operating points. These experiments nevertheless characterize only controlled static and finite-horizon sensitivity. They do not establish behavior under explicit vehicle placement, mobility-driven neighborhood changes, delayed or stale neighbor information, temporally correlated estimation errors, or burst-dependent CBR/BFR measurement noise.

The reported numerical gains should be interpreted specifically as MAC-layer contention-control results under the adopted common-contention-domain assumptions. Collision probability represents simultaneous modeled MAC access attempts. The reported event-message delay represents MAC access delay conditional on successful transmission rather than unconditional or end-to-end communication latency. Unsuccessful event-message outcomes are quantified separately through the reported collision-loss, replacement, expiration, and incomplete-transmission rates, while SBR is a transmitter-side completion metric rather than a receiver-specific PDR. Broadcast transmissions are modeled without acknowledgement-triggered retransmission. Hidden-terminal relationships, mobility-dependent topology, distance-dependent propagation loss, time-varying fading, receiver capture, external interference, and detailed physical-layer decoding are outside the present model; consequently, the reported numerical improvements should not be extrapolated directly to receiver-side packet delivery or to deployment-level vehicular communication reliability. Accordingly, the present evaluation should be interpreted as verification of the proposed MAC-layer contention-control logic rather than as validation of receiver-side vehicular communication reliability.

The quantitative results also depend on the adopted traffic, queueing, and evaluation assumptions. Periodic traffic is generated at 10 Hz, event traffic follows the adopted hyperexponential/EventScale model, each vehicle retains a single pending safety message, newer messages may replace older ones, and a 50 ms validity deadline is imposed. Under the primary evaluation considered here, the deadline-expiration outcome was not observed; incomplete event-message transmission arose from collision loss and message replacement. In addition, the primary evaluation is a structured two-factor grid in which vehicle population and event-message load vary while the remaining networking and MAC parameters are held fixed. The aggregate results therefore characterize consistency across the evaluated density–traffic operating points and should not be interpreted as evidence of invariance across arbitrary vehicular-network configurations.

Overall, the results support the use of multi-indicator contention estimation when a fixed contention window is insufficient but aggressive density-driven expansion is undesirable. MIRA-EDCA combines potential contention, realized channel activity, and node-level backoff interruption within a bounded and interpretable control rule, while retaining the class-specific priority structure of the underlying contention mechanism. The method should therefore be viewed as a bounded and interpretable multi-indicator contention-control alternative for the evaluated IEEE 802.11p safety-broadcasting conditions, rather than as a universal replacement for density-driven, learning-based, or more complete cooperative MAC protocols.

Future work will examine whether the observed contention-control behavior is retained under mobility-dependent topology, realistic propagation and fading, hidden-terminal effects, receiver capture, external interference, receiver-specific packet reception, and physical-layer decoding. Such extensions will enable receiver-side Packet Delivery Ratio and end-to-end safety-message reception to be evaluated separately from the transmitter-side metrics considered in the present study. Future work will also investigate practical local-contender estimation from periodically received neighbor information and assess the influence of delayed, biased, or temporally correlated estimates on contention-window adaptation.

## 5. Conclusions

This paper proposed MIRA and integrated it with the class-specific priority-access structure of EDCA, forming the MIRA-EDCA contention-window adaptation mechanism for IEEE 802.11p vehicular safety broadcasting. MIRA combines the normalized local contender indicator, CBR, and BFR into a bounded contention-pressure measure, while MIRA-EDCA applies the resulting CW adaptation separately to event-driven and periodic safety traffic without removing their EDCA-based priority differentiation.

The proposed method was evaluated over 30 operating points of a structured two-factor density–traffic grid, formed by ten vehicle-population levels and three event-load levels, with 30 Monte Carlo realizations per operating point, and compared with Standard EDCA, the implemented DYCW-2023-CE proxy, and the frozen MA-DRL-2026-CE-PS learning-based proxy. Across the primary density–traffic grid, MIRA-EDCA reduced the mean collision probability from 0.008407 for Standard EDCA to 0.005724, corresponding to a 31.92% reduction, while increasing the conditional mean MAC access delay of successfully transmitted event messages by only 0.66%, from 0.3341 ms to 0.3363 ms. For *N* ≥ 100, the collision reduction reached 33.40%, while the corresponding conditional mean-delay increase remained below 1%.

Under the implemented proxy comparisons and matched evaluation assumptions, MIRA-EDCA reduced the overall conditional mean event-message MAC access delay by approximately 88.6% relative to DYCW-2023-CE and by approximately 31.7% relative to MA-DRL-2026-CE-PS. The corresponding conditional median and 95th-percentile delays were 0.3182 and 0.4837 ms for MIRA-EDCA, compared with 2.9246 and 5.4286 ms for DYCW-2023-CE and 0.3657 and 1.0293 ms for MA-DRL-2026-CE-PS. Although the learning-based proxy had a slightly higher overall mean transmitter-side SBR than MIRA-EDCA (0.992378 versus 0.992009), none of the 30 operating-point SBR differences remained statistically significant after Holm–Bonferroni correction. These comparisons characterize only the implemented contention-window control proxies under the adopted evaluation assumptions and do not establish general superiority over the complete source methods.

Because the reported MAC access-delay statistics are conditional on successful event-message transmission, unsuccessful outcomes were additionally quantified separately. Across the 30 operating points, the incomplete-event rates were 0.8344% for Standard EDCA, 1.6985% for DYCW-2023-CE, 0.7991% for MIRA-EDCA, and 0.7622% for MA-DRL-2026-CE-PS. No event-message expiration or residual pending event was observed under the evaluated 50 ms validity and drain rules. The replacement rates were 0.0285%, 1.1013%, 0.0292%, and 0.0900%, respectively, with the remaining incomplete outcomes arising from modeled collision loss. Thus, the conditional delay results are interpreted jointly with terminal-outcome rates rather than as unconditional latency measurements for all generated event messages.

The ablation study further clarified the roles of the three contention observations used by MIRA. Density-only adaptation reduced collision probability but increased the mean active event CW state count to approximately 20.56 and increased event-message MAC access delay by approximately 38.15% relative to Standard EDCA. Under the frozen baseline quantization, the Density + CBR Joint-Load formulation remained numerically equivalent to Standard EDCA because its contention-pressure values did not cross the first CW quantization boundary sufficiently often to change the active event-CW state.

The additional quantization-sensitivity experiment showed that this equivalence is not a structural property of the Density + CBR formulation. When the first contention-pressure transition threshold was reduced from approximately 0.333 to 0.20 without changing the Joint-Load expression or the remaining controller settings, Joint-Load left the baseline event-CW state at 5 of the 30 operating points and its mean event CW state count increased from 4.000 to 4.032. Its overall mean collision probability changed from 0.008407 to 0.008364, while the mean event-message MAC access delay changed from 0.33412 to 0.33443 ms.

The complete MIRA formulation remained substantially more responsive across the evaluated operating space. MIRA-EDCA left the baseline event-CW state at all 30 operating points under both quantization structures, while its aggregate collision–delay operating point changed only slightly: the mean collision probability changed from 0.005724 to 0.005762 and the mean event-message MAC access delay from 0.33634 to 0.33629 ms. These results indicate that BFR should not be interpreted as universally necessary for Density + CBR-based CW adaptation; rather, in the frozen MIRA controller, it provides complementary node-level backoff information that enables broader CW adaptation under the adopted quantization structure.

The sensitivity experiments further characterized the dependence of the frozen MIRA-EDCA controller on the tested parameter and input perturbations. A −20% static bias in the nominal contender-count input changed the mean collision probability by approximately +2.59%, whereas a +20% bias changed it by approximately −1.55%. Adaptation-epoch duration produced a stronger response, with the 50 ms setting increasing mean collision probability by approximately 42.5% relative to the frozen 100 ms baseline. The measurement-horizon experiment did not reveal a statistically significant 60 s versus 15 s difference in collision probability or event-message MAC access delay across the six evaluated sensitivity operating points. These results characterize only the tested static and finite-horizon perturbations and do not establish robustness to delayed, time-correlated, burst-dependent, or small-sample measurement errors. Accordingly, no claim of practical deployability is made on the basis of the present sensitivity analysis.

Overall, the results indicate that jointly representing potential contention, aggregate channel occupancy, and local backoff interruption can shift the evaluated MAC-layer contention operating point toward lower collision probability while keeping the conditional access delay of successfully transmitted event messages close to Standard EDCA. The deterministic bounded-fusion and quantized one-step adaptation structure provides an interpretable relationship between the observed contention state and the resulting CW state-count adjustment.

Accordingly, the conclusions of this study are limited to MAC-layer contention-control behavior under the adopted common-contention-domain assumptions. Collision probability refers to modeled simultaneous MAC access attempts, while the reported event-message latency is MAC access delay conditional on successful transmission; unsuccessful event-message outcomes are reported separately through collision-loss, replacement, expiration, residual, and incomplete-transmission rates. The reported SBR remains a transmitter-side completion metric rather than a receiver-specific Packet Delivery Ratio. The numerical results should therefore not be extrapolated directly to receiver-side packet delivery or deployment-level reliability under hidden terminals, mobility-dependent topology, propagation loss, fading, receiver capture, external interference, or detailed physical-layer decoding. Future validation should examine whether the observed contention-control behavior is retained when these channel, topology, and reception effects are introduced.

## Figures and Tables

**Table 1:** Positioning of representative vehicular MAC and contention-control approaches relative to the MIRA-EDCA design.

Method	Potential Contention Information	Realized Channel/Load Information	Node-Level Backoff Interruption	Priority Support	Training Required	Main Control Characteristic Relative to MIRA-EDCA
Standard EDCA [1]	No adaptive contender estimate	No adaptive load indicator	Backoff freezing is part of operation but not used for CW adaptation	Yes	No	Fixed class-specific CW/AIFS
Yu et al. [13]	Vehicle density	Network busy factor	Not explicitly used as a separate adaptation indicator	Limited	No	Density-derived initial CW with busy-factor adjustment
Chien and Giang [14]	Indirect	Successful sent/received frame statistics	Not explicitly isolated	Yes	No	Feedback-driven CW adjustment
ASCW [15]	Indirect	Successful-frame percentage	Not explicitly isolated	Yes	No	Sliding CW with persistence factor
Context-aware MAC [16]	Vehicular/context information	Not primary	Not explicitly isolated	Yes	No	Priority/context-based CW adaptation
DYCW [17]	Vehicle density	No explicit load term in the density rule	No explicit BFR term	Not explicit in density rule	No	Density-range-to-CW mapping
ACW [18]	Contender count	Collision-related information	No explicit BFR term	Not explicit	No	Analytical/threshold-based CW selection
Q-learning [19]	Collective contention estimate	State-dependent	Not separately isolated as a deterministic indicator	Not central	Yes	Learned channel-access policy
Pei et al. [20]	Two-hop neighbor count/vehicle-density information	No separate CBR-like load term	No explicit BFR term	Beacon/emergency traffic differentiated	Yes	Q-learning-based CW selection combined with multi-hop emergency broadcasting
Actor–Critic CW [21]	Environment state	State-dependent	Not separately isolated as a deterministic indicator	Not central	Yes	Learned CW selection
Kim et al. [22]	Vehicle density and traffic demand	CCH/SCH traffic-load information	No explicit BFR term	Not central to CW rule	Yes	RL-based CW adjustment with adaptive CCHI/SCHI resource allocation
MA-DRL CW [23]	Local MAC observations	Included in local state	Not isolated as the MIRA BFR component	Not central	Yes	Multi-agent learned CW policy
CFC-MAC [24]	Neighbor/vehicle and trajectory information	Occupied/free slot and collision-related information	Not applicable to contention-free access	Not central	No	Cooperative contention-free slot allocation and heterogeneous-collision resolution
**MIRA-EDCA**	**Normalized local contender indicator**	**Channel Busy Ratio**	**Backoff Freeze Ratio**	**Yes**	**No**	**Bounded deterministic multi-indicator fusion with class-specific quantization and one-level-per-epoch CW transition**

**Table 2:** Simulation parameters used in the evaluation.

Parameter	Value/Range
Network scenario	Local IEEE 802.11p V2V contention domain
Vehicle mobility	Not explicitly modeled
Road length, (*L*)	2000 m
Communication range, (*R*)	300 m
Global number of vehicles, (*N*)	{20, 40, …, 200}
Local population	Equation (1)
Slot duration, (*σ*)	13 μs
SIFS	32 μs
Transmission duration, (*T_tx_*)	254 μs
Event AIFSN	2
Periodic AIFSN	3
Standard/base event CW state count, *W_E_*	4
Standard/base periodic CW state count, *W_P_*	8
MIRA-EDCA event CW state-count levels	{4, 8, 16, 32, 64, 128}
MIRA-EDCA periodic CW state-count levels	{8, 16, 32, 64, 128}
Reference contender-count parameter, *W_ref_*	8
Baseline adaptation epoch	100 ms
Periodic message rate	10 Hz/vehicle
Hyperexponential probabilities	*p*_1_ = 0.8, *p*_2_ = 0.2
Event rates	*λ*_1_ = 0.5 *s*^−1^, *λ*_2_ = 5 *s*^−1^
EventScale	{1, 3, 5}
Message validity deadline	50 ms
Main measurement interval	15 s
Drain interval	50 ms
Main Monte Carlo realizations	30/operating point
Main density–traffic operating points	30
Main seed base	710,000

Note: All CW-related numerical values in Table 2 are state counts W. The corresponding conventional maximum backoff index is CW_conv_ = *W* − 1; for example, *W* = 4 corresponds to CW_conv_ = 3, and *W* = 8 corresponds to CW_conv_ = 7.

**Table 3:** Fidelity and scope of the benchmark implementations used in the common evaluation environment.

Comparator	Source Basis	Component Reproduced or Transferred	Common-Environment Adaptation	Source Components Not Reproduced	Interpretation in This Study
**Standard EDCA**	IEEE 802.11p/EDCA reference configuration [1]	Fixed class-specific contention-window and AIFS-based priority behavior	Implemented in the same slot-level traffic and contention environment used for all methods	Full physical-layer and deployment-specific IEEE 802.11p behavior outside the modeled contention process	Fixed contention-window reference
**DYCW-2023-CE**	Density-driven dynamic contention-window rule in [17]	Published mapping from surrounding vehicle population to discrete CW values	The source density variable is mapped to the effective local contender population *N_eff_*; the rule is evaluated under the same traffic, backoff, and channel-access assumptions as the other methods	Cooperative communication mechanisms and other source-specific protocol components outside the published density-to-CW rule	Common-environment proxy for density-driven CW control; not an exact reproduction of the complete method in [17]
**MA-DRL-2026-CE-PS**	Learning-based CW adaptation direction represented by [23]	Actor–Critic-based CW control using locally observable MAC-related state information	Implemented as a parameter-shared learning controller and frozen before held-out evaluation; the same traffic and channel-access environment is used as for the other methods	Source-specific implementation details that are not sufficiently specified for exact reproduction	Common-environment learning proxy; not an exact reproduction of the complete method in [23]

**Table 4:** Summary of high-density collision–access-delay operating points for the evaluated contention-control methods (N ≥ 100).

Method	Mean Collision	Conditional Mean Event Delay (ms)	Mean Transmitter-Side Event SBR
Standard EDCA	0.012080 ± 0.000658	0.34179 ± 0.00022	0.988228 ± 0.000368
DYCW-2023-CE	0.008305 ± 0.000116	4.2774 ± 0.0057	0.975425 ± 0.000449
MIRA-EDCA	0.008046 ± 0.000362	0.34517 ± 0.00023	0.988857 ± 0.000408
MA-DRL-2026-CE-PS	0.008375 ± 0.000291	0.4634 ± 0.0013	0.989786 ± 0.000313

**Table 5:** Overall performance summary of the MIRA-EDCA ablation variants across the 30 operating points of the primary density–traffic grid.

Configuration	Mean Collision Probability	Mean Transmitter-Side Event SBR	Mean Event MAC Access Delay (ms)	Mean Event CW State Count
Standard EDCA	0.008407 ± 0.000490	0.991656 ± 0.000219	0.33412 ± 0.00019	4.000 ± 0.0
Density-only	0.006402 ± 0.000219	0.992478 ± 0.000183	0.46160 ± 0.00037	20.562 ± 0.0
Joint-Load	0.008407 ± 0.000490	0.991656 ± 0.000219	0.33412 ± 0.00019	4.000 ± 0.0
MIRA-EDCA	0.005724 ± 0.000286	0.992009 ± 0.000271	0.33634 ± 0.00018	4.269 ± 0.012

**Table 6:** Quantization-sensitivity analysis of the Joint-Load and complete MIRA-EDCA formulations across the 30 primary density–traffic operating points.

Quantization	Method	Mean Collision Probability	Mean Event SBR	Mean Event MAC Access Delay (ms)	Mean Event CW State Count	Operating Points with W*_E_* > 4
Baseline-Q	Standard EDCA	0.008407	0.991656	0.33412	4.000	0/30
Baseline-Q	Joint-Load	0.008407	0.991656	0.33412	4.000	0/30
Refined-Q	Joint-Load	0.008364	0.991702	0.33443	4.032	5/30
Baseline-Q	MIRA-EDCA	0.005724	0.992009	0.33634	4.269	30/30
Refined-Q	MIRA-EDCA	0.005762	0.991995	0.33629	4.263	30/30

**Table 7:** Sensitivity of MIRA-EDCA to the reference contender-count parameter *W_ref_* and static bias in the nominal local contender-count input.

Configuration	Mean Collision Probability	Mean Transmitter-Side Event SBR	Mean Event MAC Access Delay (ms)	Mean Event CW State Count
*W_ref_* = 4	0.008356 ± 0.000541	0.987668 ± 0.000792	0.34699 ± 0.00034	4.502 ± 0.042
*W_ref_* = 8	0.008705 ± 0.000563	0.987480 ± 0.000745	0.34644 ± 0.00033	4.427 ± 0.034
*W_ref_* = 16	0.009214 ± 0.000608	0.987554 ± 0.000805	0.34575 ± 0.00038	4.333 ± 0.025
$\hat{N}$ − 20%	0.008930 ± 0.000566	0.987432 ± 0.000769	0.34623 ± 0.00033	4.399 ± 0.032
$\hat{N}$ + 20%	0.008570 ± 0.000569	0.987611 ± 0.000729	0.34660 ± 0.00034	4.450 ± 0.037

**Table 8:** Sensitivity of MIRA-EDCA performance to the measurement duration. Values are reported as mean ± 95% confidence-interval half-width over 20 Monte Carlo realizations after averaging the six sensitivity operating points, *N* = {100, 160, 200}, EventScale = {1, 5}.

Measurement Time *T_meas_*(s)	Mean Collision Probability	Transmitter-Side Event SBR	Event-Message MAC Access Delay (ms)	Mean Event CW State Count
15	0.008705 ± 0.000563	0.987480 ± 0.000745	0.34644 ± 0.00033	4.427 ± 0.034
30	0.007529 ± 0.000451	0.988766 ± 0.000431	0.34586 ± 0.00054	4.392 ± 0.030
60	0.008104 ± 0.000638	0.988401 ± 0.000299	0.34636 ± 0.00049	4.416 ± 0.041

**Table 9:** Association between CBR and BFR for the frozen MIRA-EDCA realizations of the primary density–traffic evaluation.

Analysis Scope	*n*	Pearson *r*	95% CI	Spearman *ρ*	*R* ^2^
All primary MIRA-EDCA realizations	900	0.720	0.687–0.750	0.767	0.518
High density, *N* ≥ 100	540	0.626	0.571–0.674	0.608	0.391
Within operating point, demeaned	900	−0.159	−0.222–−0.095	−0.162	0.025

Note: The within-operating-point analysis removes the mean CBR and BFR associated with each (*N*, EventScale) operating point before calculating the correlation. It therefore characterizes residual association after controlling for the common density–traffic condition.

## Data Availability

The simulation-generated data and supporting implementation materials are available from the corresponding author upon reasonable request.
